# Instability of circular RNAs in clinical tissue samples impairs their reliable expression analysis using RT-qPCR: from the myth of their advantage as biomarkers to reality

**DOI:** 10.7150/thno.46341

**Published:** 2020-07-23

**Authors:** Hannah Rochow, Antonia Franz, Monika Jung, Sabine Weickmann, Bernhard Ralla, Ergin Kilic, Carsten Stephan, Annika Fendler, Klaus Jung

**Affiliations:** 1 Department of Urology, Charité - University Medicine, 10117 Berlin, Germany.; 2 Berlin Institute for Urologic Research, 10115 Berlin, Germany.; 3 Department of Pathology, Charité - University Medicine, 10117 Berlin, Germany.; 4 Institute of Pathology, Hospital Leverkusen, 51375 Leverkusen, Germany.; 5 Max Delbrueck Center for Molecular Medicine in the Helmholtz Association, Cancer Research Program, 13125 Berlin, Germany.; 6 Cancer Dynamics Laboratory, The Francis Crick Institute, 1 Midland Road, London NW1 1AT, U.K.

**Keywords:** circular RNAs, RNA integrity, RNA degradation, circRNA stability, normalization, RT-qPCR

## Abstract

**Background:** Circular RNAs (circRNAs) are a new class of RNAs with medical significance. Compared to that of linear mRNA transcripts, the stability of circRNAs against degradation owing to their circular structure is considered advantageous for their use as biomarkers. As systematic studies on the stability of circRNAs depending on the RNA integrity, determined as RNA integrity number (RIN), in clinical tissue samples are lacking, we have investigated this aspect in the present study under model and clinical conditions.

**Methods:** Total RNA isolated from kidney cancer tissue and cell lines (A-498 and HEK-293) with different RIN after thermal degradation was used in model experiments. Further, RNA isolated from kidney cancer and prostate cancer tissue collected under routine surgical conditions, representing clinical samples with RIN ranging from 2 to 9, were examined. Quantitative real-time reverse-transcription polymerase chain reaction (RT-qPCR) analysis of several circRNAs (*circEGLN3, circRHOBTB3, circCSNK1G3, circRNA4*, and *circRNA9*), their corresponding linear counterparts, tissue-specific reference genes, and three microRNAs (as controls) was performed. The quantification cycles were converted into relative quantities and normalized to the expression of specific reference genes for the corresponding tissue. The effect of RIN on the expression of different RNA entities was determined using linear regression analysis, and clinical samples were classified into two groups based on RIN greater or lesser than 6.

**Results:** The results of model experiments and clinical sample analyses showed that all relative circRNA expression gradually decreased with reduction in RIN values. The adverse effect of RIN was partially compensated after normalizing the data and limiting the samples to only those with RIN values > 6.

**Conclusions:** Our results suggested that circRNAs are not stable in clinical tissue samples, but are subjected to degradative processes similar to mRNAs. This has not been investigated extensively in circRNA expression studies, and hence must be considered in future for obtaining reliable circRNA expression data. This can be achieved by applying the principles commonly used in mRNA expression studies.

## Introduction

Circular RNAs (circRNAs) are a new class of non-coding RNAs. These RNAs are single-stranded and have a covalently closed circular structure lacking both the 5'-cap and the characteristic 3′-poly(A) tail of linear RNA 1, 2. Although identified in the 1970s 3, they were considered trash elements without any actual biological function until 2010. Owing to the widespread application of advanced sequencing technologies and the advancements in bioinformatics, circRNAs are now known to be ubiquitously expressed and highly conserved cellular components 1, 2, 4, 5. Several studies have shown that circRNAs play important roles in the maintenance of endogenous homeostasis. In contrast, many diseases, especially cancers, are frequently accompanied by altered circRNA expression profiles in the affected tissues and body fluids 6-9. Thus, circRNAs are of particular interest as both diagnostic, prognostic, and predictive biomarkers, as well as therapeutic target structures 6, 7, 10, 11.

Meaningful prospective studies have to be performed to translate these initial promising findings of circRNA biomarker research into clinical practice 12. This requires specifying the pre-analytical and analytical requirements for reliable determination of circRNA expression. The challenges in this respect have been discussed in several reports 1, 13, 14. In a previous study, we have discussed the various analytical problems and the necessary methodological approaches for the identification, validation, and quantification of circRNAs 12.

In addition to high-throughput sequencing, hybridization, and microarray approaches used for the detection circRNAs and expression profiling in samples, quantitative real-time reverse-transcription polymerase chain reaction (RT-qPCR) is an indispensable tool for circRNA research. This approach is essential for validation of circRNAs that have been identified in genome-wide screening using sequencing or microarray technology 4, 15, 16. Furthermore, RT-qPCR can be used for quantifying the expression of single circRNAs conveniently when their validity as a biomarker has to be assessed in clinical studies 7. However, RT-qPCR is error-prone owing to variations in the quality of the RNA used, in particular the integrity of the total RNA samples under investigation 17-19. Unfavorable sample collection procedures under clinical conditions are a common pre-analytical explanation for the degradation of isolated total RNA 20. For mRNAs, numerous studies have confirmed that an RNA integrity number (RIN) of <5 is indicative of total RNA degradation, which is associated with reduction in relative mRNA quantities 17-19, 21. This is much less true for miRNAs owing to their short length of 20-22 nucleotides 17, 22. Studies have shown that the normalization of mRNA expression data of degraded total RNA samples to reference genes may partially compensate for this detrimental effect of RNA integrity on expression analysis 17-19, 23. Many studies have emphasized the advantage of using circRNAs as biomarkers owing to their stability 1, 7, 24, 25. Little is known to date about the *in vivo* degradation of circRNAs 26. The intracellular stability of circRNAs is due to their unique circular structure, which renders them resistant to exonucleases such as RNase R 27. This does not consider though the homeostasis of the complex RNA degradation pathways, which can be perturbed during the collection of clinical tissue samples before they are stored under stabilizing conditions 28, 29. This might also change the potential degrading activity of endonucleases on total RNA under these conditions. Some studies have noted potential difficulties in quantifying circRNAs in degraded total RNA samples 7, 13, 30, 31. Therefore, it is surprising that the majority of recent circRNA expression-related studies in cancer tissues have ignored the possible influence of RIN on the measured values (Table S1). Studies in peripheral blood samples considered this effect 32-35. Overall, systematic studies on circRNAs, similar to those mentioned above for mRNAs, are lacking, although they are necessary. In the following, the term stability is exclusively used to characterize the relationship between the expression values of circRNAs or mRNAs measured by RT-qPCR and the RNA integrity of the test samples.

Therefore, the aims of this study were to investigate (a) how RNA integrity, as a pre-analytical factor, affects the RT-qPCR results of circRNAs, (b) whether the stability of circRNAs differs from those of their linear counterparts, tissue-specific reference genes, and miRNAs, and (c) whether the adverse effects of poor total RNA integrity on the RT-qPCR results of circRNAs can be corrected or reduced by normalizing with the expression of reference genes. Toward these objectives, we first performed model experiments, in which we determined the expression of different RNA species in artificially degraded total RNA samples isolated from two cell lines and a kidney cancer tissue pool. Subsequently, we addressed the clinical situation by determining RIN-dependent expression of different RNAs in total RNA isolated from kidney and prostate cancer tissue specimens. In clinical tissue samples, we examined three circRNAs (Table 1) that are annotated in the database circBAse as *hsa_circ_0101692, hsa_circ_0001522*, and *hsa_circ_0007444* 36. The first two are validated circRNAs in the kidney or prostate; the third was detected in both organs 37, 38. Furthermore, the linear transcripts of the host genes of the circRNAs were measured. In the following, the terms *circEGLN3, circCSNK1G3*, and *circRHOBTB3* for the circRNAs and *linEGLN3, linCSNK1G3*, and *linRHOBTB3* for the linear counterparts are used in reference to the corresponding host genes to facilitate the presentation of comparisons (Table 1).

## Materials and Methods

### Tissue specimens and cell lines

Tissue samples were obtained from patients with clear cell renal cell carcinoma (ccRCC) and prostate cancer (PCa) undergoing radical nephrectomy and radical prostatectomy, respectively. The Ethics Committee of the Charité - University Medicine, Berlin, approved the study (EA1/135/12) and informed consent was obtained from all patients. The study was performed in accordance with the Declaration of Helsinki. The clinicopathological characteristics of the patients are summarized in Tables S2 and S3. The samples were randomly selected from our RNA isolation bank, but were limited by RNA amount and the completeness of patient data. Tissue samples were snap-frozen in liquid nitrogen immediately after surgery and stored at -80 °C or transferred into the RNAlater stabilization reagent (Qiagen, Hilden, Germany) and stored at -20 °C until RNA isolation as described previously 39, 40. The human cell lines A-498 (ATCC no. HTB-44; established from human kidney carcinoma) and HEK-293 (ATCC CRL-1573; established from human embryonic kidney) were cultured under standard conditions and harvested at 80-90% confluence.

### Extraction and quality control of total RNA

Total RNA, including miRNAs, was isolated using the miRNeasy mini kit (Qiagen) as described previously 22, 39-41. Briefly, approximately 50 mg tissue or 1 × 10^6^ cells were disrupted in 700 µL Qiazol in a TissueLyser (Qiagen) at 30 Hz for 2 × 1 min. The homogenate was processed according to the manufacturer's instructions, with the inclusion of an on-column DNase digestion step. RNA was eluted from the spin column membrane with 30 µL nuclease-free water. RNA yield and purity were controlled by measuring the absorbance on the NanoDrop ND-1000 spectrophotometer (NanoDrop Technologies, Wilmington, DE, USA). The median RNA concentrations in the tested clinical samples were 1086 (95% CI: 996 to 1172) ng/µL from kidney cancer and 957 (95% CI: 846 to 1031) ng/µL from prostate cancer. The ratio of the absorbance at 260 nm and 280 nm of all isolated RNA samples ranged from 1.89 to 2.01. The RIN was assessed on a Bioanalyzer 2100 with the Agilent RNA 6000 Nano Chip Kit (Agilent Technologies, Santa Clara, CA, USA; Cat. No. 5067-1511). Isolated RNA samples were stored at -80 °C until analysis. Further details are listed in the checklist (Table S4) of the Minimum Information for Publication of Quantitative Real-Time PCR (MIQE) guidelines 42.

### *In vitro* RNA thermal degradation experiments

**Figure 1:**
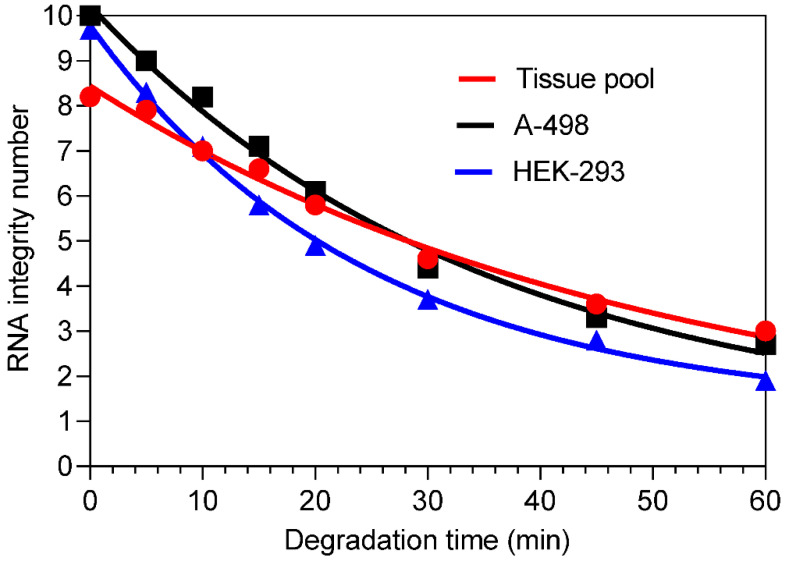
Decrease in RNA integrity after time-dependent thermal degradation of total RNA isolated from renal cell lines A-498 and HEK-293, and the kidney cancer tissue pool. The RNA samples were incubated at 80 °C in Eppendorf tubes for different time points and subsequently transferred to an ice-bath to stop the degradation and stored at -80 °C until analysis. The RIN values at the time points were for the A-498 cells: 10.0, 9.0, 8.2, 7.1, 6.1, 4.4, 3.3, and 2.7; for the HEK-293 cells: 9.7, 8.3, 7.1, 5.8, 4.9, 3.7, 2.8, and 1.9 (the last sample was excluded in further analysis); and for the tissue pool: 8.2, 7.9, 7.0, 6.6, 5.8, 4.6, 3.6, and 3.0. The gel like view of total RNA samples after heat degradation analyzed with the total Agilent RNA 6000 Nano Chip Kit on the Agilent 2100 bioanalyzer is shown in Figure S1.

**Figure 2:**
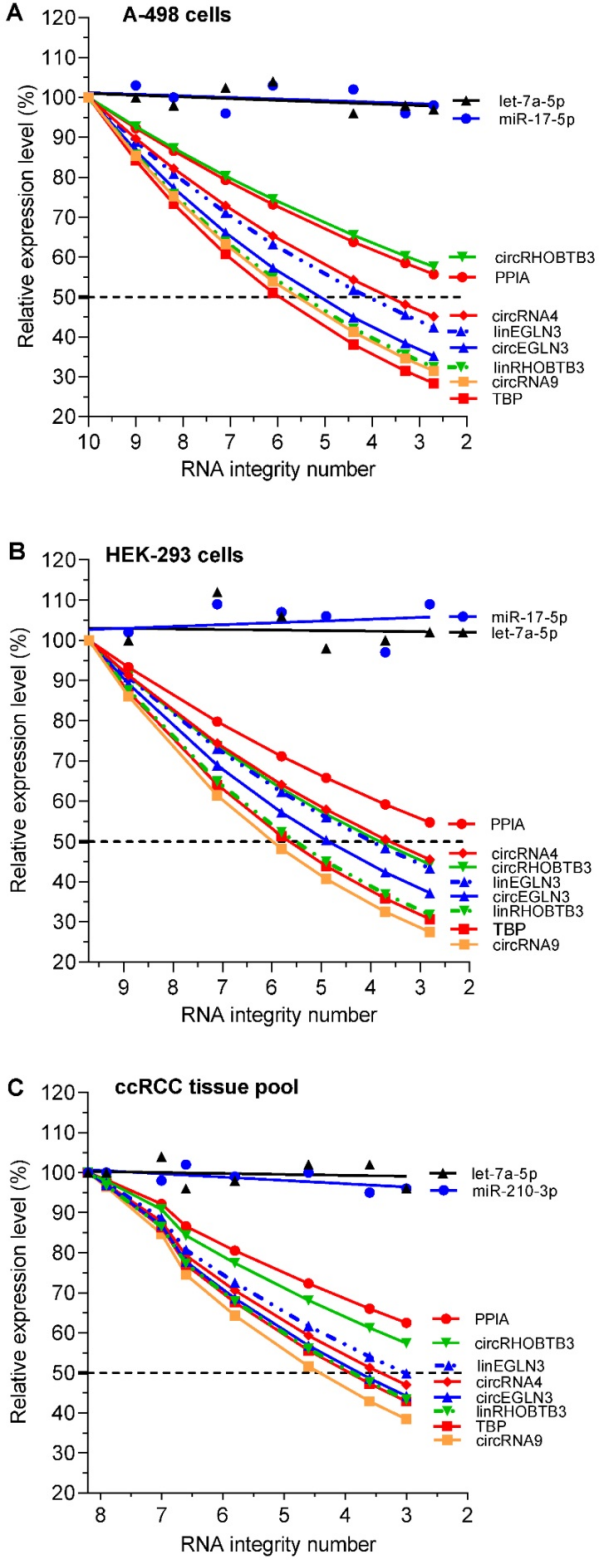
Expression of circRNAs, mRNAs, and miRNAs depending on RNA degradation in renal cell lines A-498 and HEK-293, and the kidney cancer tissue pool. In the RNA samples shown in Figure 1 with their RNA integrity number (RIN) listed in its legend, all RNA variables (including the conventional reference genes *PPIA* and *TBP* for mRNA expression studies in kidney cancer) were analyzed using RT-qPCR and converted to relative values, which are depicted as percentage mean values of triplicates with respect to the starting RIN (RIN 10 for A-498, 9.7 for HEK-293, and 8.2 for kidney cancer tissue pool). In order not to impair the clarity of the figure with the various curves, no error bars were drawn in. The analytical variations of the intra-plate measurements for the RNA variables in the degradation experiments correspond to the %RSD values of the repeatability data in Table S11 (<9%). Linear regression lines were calculated for miRNAs with slopes that did not differ significantly from zero (*P-*values between 0.157 and 0.877) and with intercepts between 94% and 107% with 95% confidence intervals, while always including the starting point of 100%. *CircRNA4* and *circRNA9* were included in this study as controls to confirm the effect of RNA integrity on established circRNAs used in other studies 4. Abbreviations: *PPIA:* peptidylprolyl isomerase A; *TBP*: tata-box binding protein.

After preliminary experiments on artificial heat degradation of total RNA samples, an experimental design was developed. This should take into account all influencing variables and allow measurement of all RNA variables under equal conditions for a representative experiment with matched samples (Figure 1 and 2). Therefore, RNA pools were prepared using total RNA samples isolated from three separate cell culture experiments and eight different ccRCC tissue samples. The RNA extracts were adjusted to equal concentrations of 650 ng/µL. The pools were prepared by mixing equal volumes of the individual RNA samples to maximally compensate for variabilities in individual expression. Ten microliters of the described RNA pools from cell lines and tissue samples were incubated in microcentrifuge tubes at 80 °C for 90 min in a thermal block cycler (Biometra GmbH, Göttingen, Germany) as described previously 22. The degradation was stopped by transferring the tubes to an ice bath. The samples were stored at -80 °C until analysis (one RIN determination with Agilent gel electrophoreses shown in Figure S1; triplicates for RNA variables).

### RT-qPCR measurements of circRNAs, mRNAs, and miRNAs

The quantification characteristics are listed in the checklist of the MIQE guidelines as mentioned above (Table S4). Detailed validation results of *circEGLN3* and *circRHOBTB3,* based on the general characteristics of circRNAs regarding their resistance to the RNase R digestion, their lack of a poly(A) tail, the amplification results in complementary DNA (cDNA) and genomic DNA (gDNA) using divergent and convergent primers, and the proof of the backsplice junctions by Sanger sequencing, are compiled in our previous report on circRNAs in kidney cancer 37. For *circCSNK1G3* measurements, the reaction conditions described in the recently published circRNA landscape of prostate cancer were used 38. The analytical specificity of the RT-qPCR products of these circRNAs were verified by melting curve analysis and gel electrophoresis (Figure S2).

#### cDNA synthesis

Maxima First Strand cDNA Synthesis Kit for RT-qPCR (Thermo Fisher Scientific, Waltham, MA, USA) including a ready-to-use mix of random hexamer and oligo(dT)_18_ primers was used for circRNAs and mRNAs in final reaction volume of 20 µL with 1 µg total RNA 37 (Table S5A). To address the issue of reliability of reverse transcription, we additionally used another cDNA synthesis kit (Transcriptor First Strand cDNA Synthesis Kit, Life Science Roche, Mannheim, Germany; Cat. No. 04379012001) that allows a separate priming with either random hexamer or oligo(dT)_18_ primers (Table S5B). The qPCR results for *circEGLN3*, *circRHOBTB3,* and *circCSNK1G3* in kidney and prostate tissue pooled samples clearly showed a marked decreased expression in all circRNAs when using oligo(dT)_18_ primers compared with hexamer primers (Figure S3). These data also prove that the circRNAs have no poly(A) tails and we can safely assume that the transcription primer mix does not impair the expression results obtained for circRNAs. On the other hand, by using a primer mix, we can ensure a maximal reverse transcription in the degraded samples for messenger RNAs that ensures that we do not impair the impact of degradation on these molecules.

Reverse transcription of miRNAs was performed with the TaqMan microRNA reverse transcription kit (Thermo Fisher Scientific, Applied Biosystems, Foster City, CA, USA) using miRNA-specific stem-looped primers according to the manufacturer's instructions, which is described in Supplementary Material, as well as in our previous reports 22, 39-41.

#### Quantification

qPCR measurements were performed on the LightCycler 480 (Roche Molecular Diagnostics, Mannheim, Germany) using white 96-well plates (Roche) in a reaction volume of 10 µL. 40 cycles were used as described previously 37. Reaction conditions, measurement details, and performance data for the circRNAs *circEGLN3, circRHOBTB3,* and *circCSNK1G3*, and their linear mRNA counterparts, the control circRNAs *circRNA4* and *circRNA9* according to Memczak et al. 4, the reference genes encoding peptidylprolyl isomerase A (*PPIA*) and TATA-box binding protein (*TBP*) for ccRCC samples 43, 5'-aminolevulinate synthase 1 (*ALAS1*) and hypoxanthine phosphoribosyltransferase 1 (*HPRT1*) for PCa samples 44, and the three miRNAs, *let‑7a‑5p, miR‑17‑5p*, and *miR‑210-3p* are summarized in Supplementary Material Tables S6-S10. No-template and no-reverse transcription controls were always performed and showed negative results. All cDNA samples were measured at least in duplicate, and the mean values of the quantification cycles (Cq) were used for calculations. To minimize analytical variation in the degradation experiments, samples of an experiment were run, as far as possible, in one plate. The repeatability of the measurements of all analytes showed variation in percentage relative standard deviations (%RSD) below 9% (Table S11). In measurements of clinical samples, run controls were used on each plate. Reproducibility of the inter-assay measurements revealed %RSD values between 7.88 and 13.8% (Table S11).

### Data analysis, statistics, and sample size calculation

Expression changes with respect to the corresponding starting points due to RNA degradation were calculated using the 2^-ΔCq^ method. The percentage changes in expression among experiments could be compared as equal amounts of total RNA were used in the RT-qPCR analyses. QBase^+^ software version 3.2 (Biogazelle, Zwijnaarde, Belgium; www.qbaseplus.com), which is based on a generalized model of the 2^-ΔΔCq^ approach with correction of amplification efficiency, was used for data evaluation 45, 46. In this program, Cq values were converted into relative quantities (RQs) with respect to the amount of total RNA (equal for all samples) used for the cDNA synthesis, and into normalized relative quantities (NRQs) based on the expression of two cancer-specific reference genes in patient samples as mentioned above.

GraphPad Prism 8.4.2 for Windows (GraphPad Software, San Diego, CA, USA) and MedCalc 19.2.0 (MedCalc Software bvba, 8400 Ostend, Belgium) were used for statistical analyses. Mann-Whitney *U*-test, Wilcoxon test, linear regression analysis, and matched analysis of variance (ANOVA) were performed as indicated in the Results. The significance of the slope was determined based on its deviation from zero and the differences between slopes. Sample size and power calculations to assess the effect of RIN on the expression of circRNAs, their linear counterparts, and reference genes in clinical tissue samples were performed using the MedCalc software. The calculation was based on comparing the mean change in the expression in two sample groups with lower (≤ 6) and higher (> 6) RIN. An effect size (difference of 1 standard deviation between the mean values of the groups with equivalent sample numbers) was considered when power was 0.9 and *P* < 0.05. Twenty-two samples were used to assess the effect of RIN under these conditions. Hence, at least 25 samples of every RIN group were included in this study. *P*-values < 0.05 (two-sided) were considered statistically significant.

## Results

### Expression of circRNAs, mRNAs, and miRNAs in total RNA degraded *in vitro*

#### *In vitro* degradation of isolated total RNA samples

To investigate the expression of circRNAs, mRNAs, and miRNAs in total RNA samples as a function of RNA integrity, the RNA samples were artificially degraded by heating. The kinetics of time-dependent RIN reduction as an indicator of changes in RNA integrity is shown in Figure 1 for RNA samples isolated from the cell lines A-498 and HEK-293, as well from the ccRCC tissue pool. An exponential one‑phase decay equation describes the effect of heat-degradation on the corresponding decreased RIN values as a function of time. This is characterized by the half-life: 24 min for A-498 cells and 17 min for HEK-293 cells. For the tissue pool, a value of 34.6 min was calculated since the initial value was already lower in comparison to the initial RIN values of RNA samples from the cell lines. However, a systematic comparison of the "decay curves" was not performed because the method of heat-degradation was only used to obtain samples of artificially degraded total RNA.

#### RT-qPCR using* in vitro* degraded RNA sample

RT-qPCR showed that the relative expression of individual RNAs after thermal treatment-mediated degradation was different from that before heating of the total RNA samples (Figure 2A-C). Collectively, these differences depended on the integrity of the RNA samples (reflected in the RIN), the source of the isolated total RNA (two cell lines and ccRCC tissue pool), the type of RNA (circRNAs, mRNAs, and miRNAs), and the individual RNAs of each RNA family. With the exception of microRNAs, which showed stable expression irrespective of the RNA integrity of the test samples, the expression of all individual circRNAs and mRNAs decreased with reduction in RIN. The degradation experiment with their dependent samples (RIN differences; different RNA variables) was evaluated with corresponding matched ANOVA calculations. The effect of the RNA integrity on the expression level of the RNA variables and also the effect between the various variables were statistically significant (*P* = 0.0055 to <0.0001). The extent of reduction in expression differed between individual circRNAs and mRNAs. For example, RNA integrity affected the expression of *TBP* mRNA and *circEGLN3* more than that of the *PPIA* mRNA and *circRHOBTB3*, respectively (Figure 2A and 2C). Furthermore, the differences in expression between circRNAs and their linear counterparts (e.g., *circRHOBTB3* vs. *linRHOBTB3*) became more evident in total RNA samples with reduced RIN. This effect partially differed depending on the source from which the total RNA was isolated.

#### Reference genes as normalizers in degraded RNA samples

**Figure 3:**
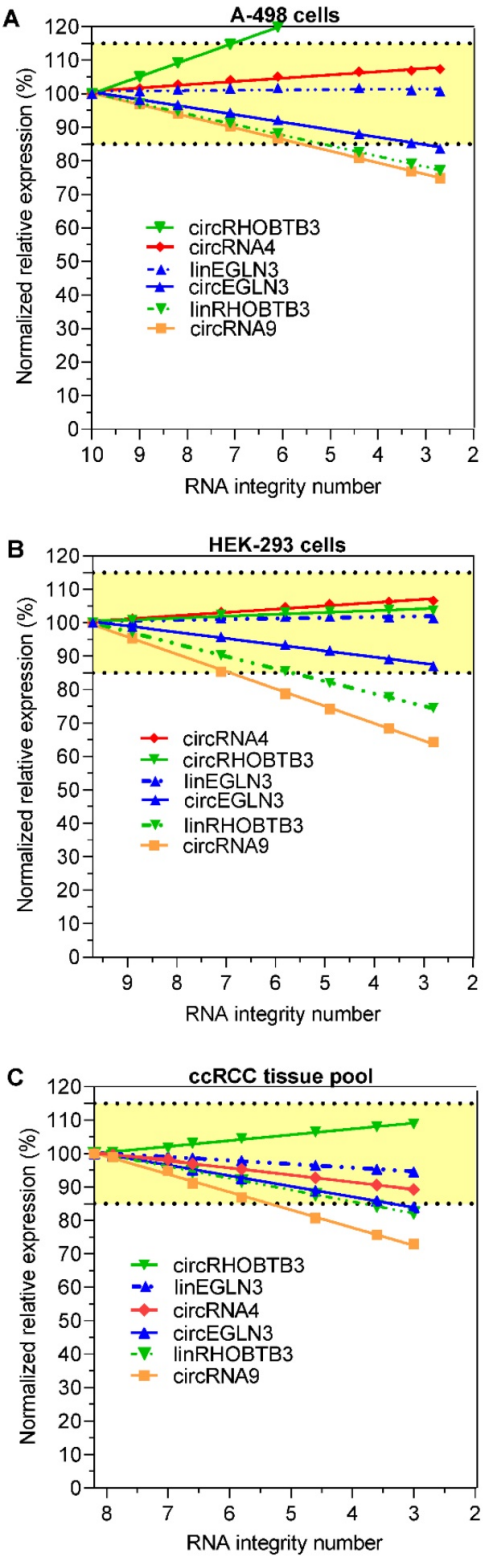
Expression of circRNAs and their linear counterparts depending on RNA degradation in renal cell lines A-498 and HEK-293, and the kidney cancer tissue pool after normalization to the reference genes *PPIA* and *TBP*. The percentage results refer to the expression data used in Figure 2, but normalized to the reference genes *PPIA* and *TBP* using the qbase^+^ software. Abbreviations: *PPIA*: peptidylprolyl isomerase A; *TBP*: tata-box binding protein.

We investigated whether the decrease in the expression of circRNAs and their respective linear transcripts in the degraded RNA samples could be compensated by normalization in order to obtain initial expression data before the degradation of the RNA samples. For this purpose, all expression data of the other RNA variables were normalized to the mean expression levels of *TBP* and *PPIA* at the respective RIN values. *TBP* and *PPIA* are exemplarily used here as validated reference genes for ccRCC expression studies 43. This approach is in agreement with the recommendations for using at least two reference genes for normalization 45-47. Figure 3A-C shows that the percentage changes in the expression of the four circRNAs and the two linear RNAs *linEGLN3* and *linRHOBTB3* after normalization to the initial expression values prior to degradation depended on the RIN of the degraded RNA samples. The percentage deviations in the expression of the degraded samples were clearly lower than the decrease in expression shown in Figure 2 due to this normalization approach. For example, the comparison of the percentage expression at RIN 6 in Figures 2 and 3 with the starting values before degradation showed that the median percentage after normalization of all six RNA variables (*circRNA4*,* circRNA9*, *circEGLN3*,* circRHOBTB3*, *linEGLN3*, and *linRHOBTB3*) in the three RNA sources amounted to 95.5% (95% CI, 88.7% to 103%), while the median percentage without this adjustment was only 65.5% (95% CI, 58 to 69.3%; n = 18, Wilcoxon test, *P* < 0.0001). These results support the view that errors due to the use of degraded RNA samples can be partially compensated via adjustment with a combination of suitable reference genes. On the other hand, over- and undercorrections of different RNAs beyond the here selected limit of 15% are observed in dependence on the RIN value of samples (Figure 3A-C). Miscorrection can occur if a different degradation pattern exists between the normalization approach and the target RNA. Thus, an additional specification of the RIN value up to which samples should be analyzed for reliable results would help to avoid this error. As recently outlined, this should be part of a multiphase process to develop circRNA assays for clinical practice 12.

### Expression of circRNAs and mRNAs in kidney and prostate cancer depends on RNA integrity

**Figure 4:**
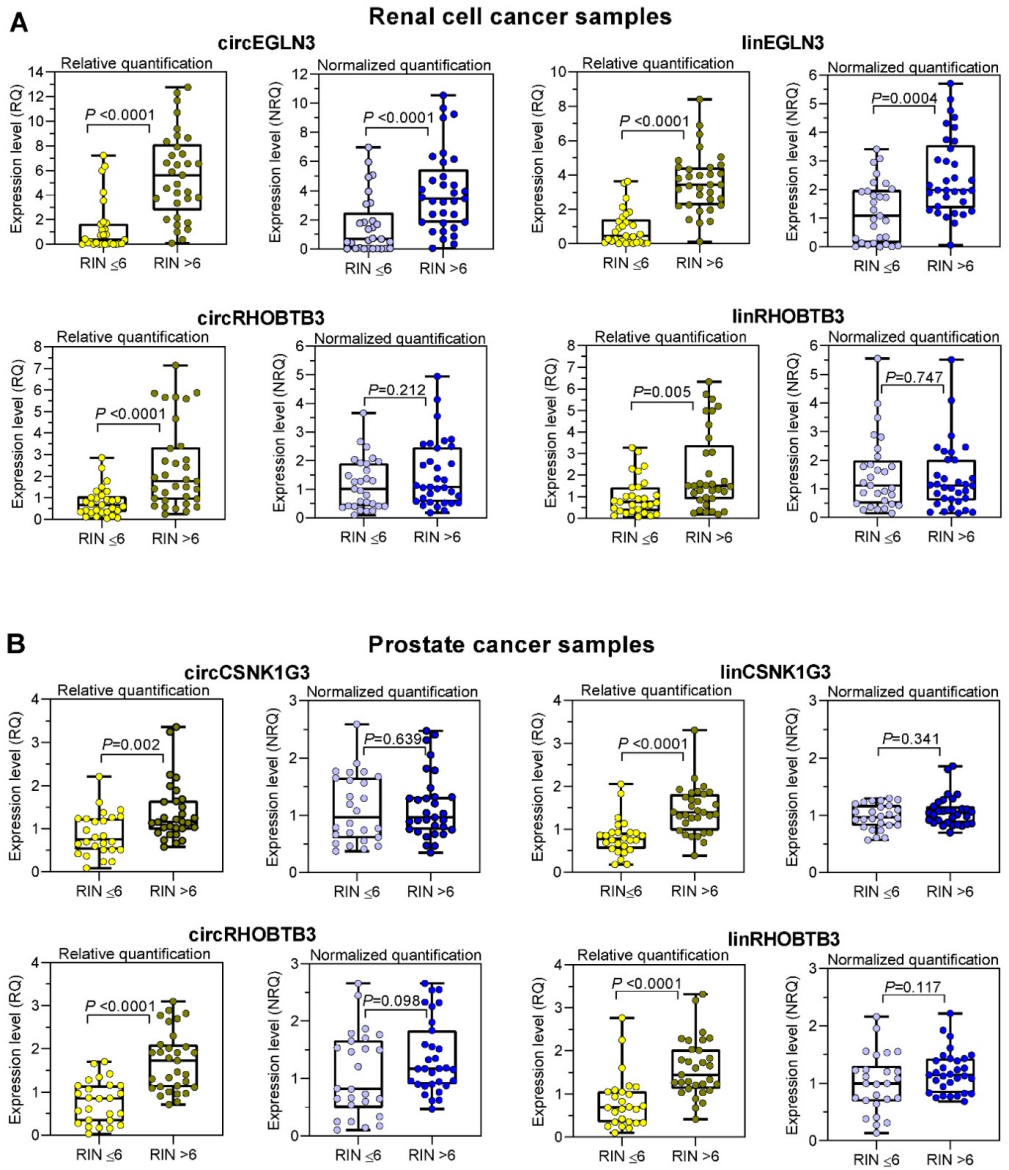
Differential expression of circRNAs and their linear counterparts in kidney and prostate cancer tissue samples with RIN values of < 6 and > 6 and calculated as relative quantities and normalized relative quantities. The expression data of RNA samples isolated from (A) clear cell renal cell carcinoma with RIN < 6 (n = 28; median 4.3, range 2.3-6.0) and > 6 (n = 33; median 7.7, range 6.1-9.4) and from (B) prostate cancer with RIN < 6 (n = 26; median 3.5, range 2.2-5.5) and > 6 (n = 31; median 7.4, range 6.3-8.2). Data are shown as box- and whisker plots with the individual values of samples. Boxes represent the lower and upper quartiles with medians; whiskers illustrate the range from the minimum to the maximum value. Expression levels are presented as relative quantities (RQs) and normalized quantities (NRQs) using the software qbase^+^ as described in Materials and Methods. Statistical significance was tested using the Mann-Whitney *U*-test.

**Figure 5:**
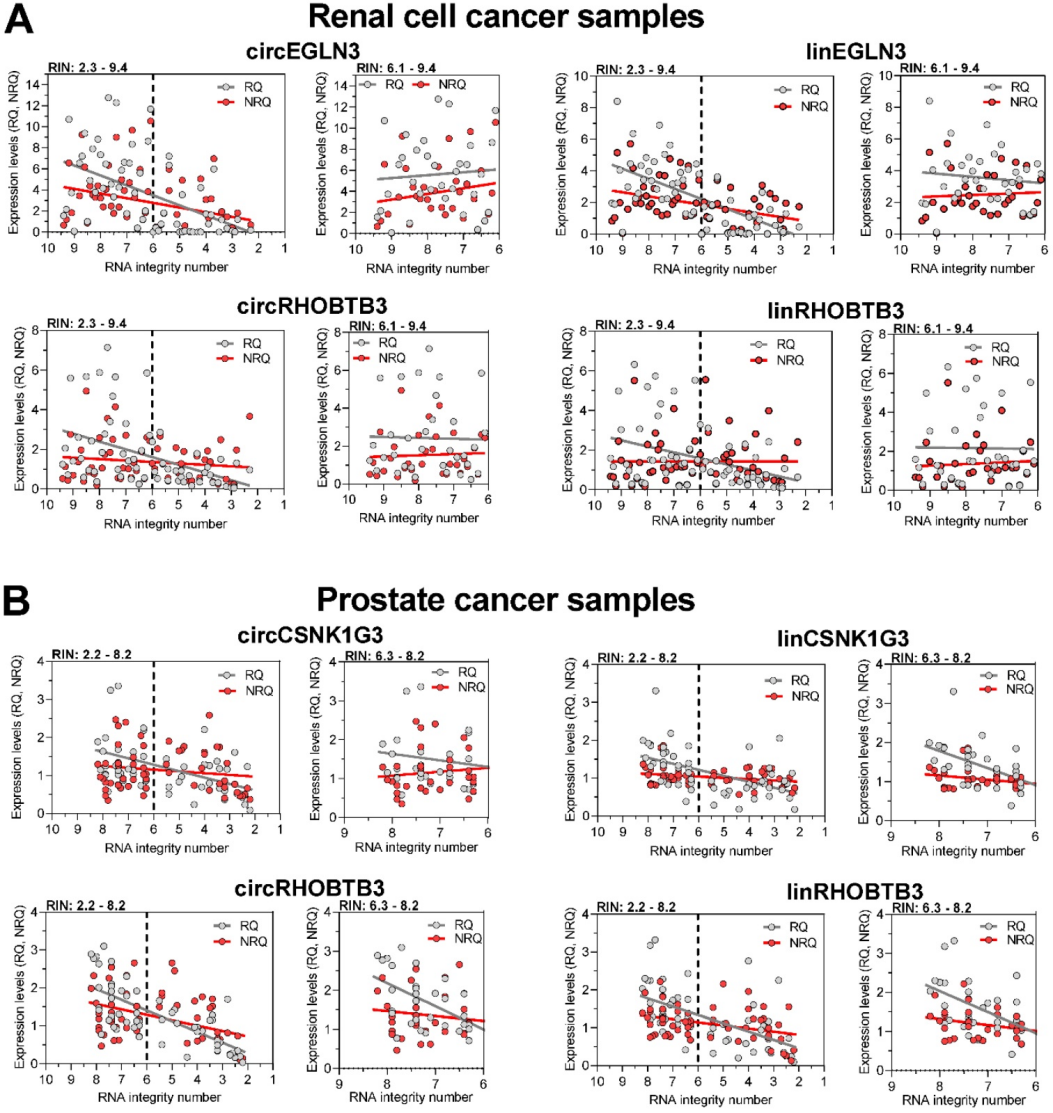
Regression analysis of the expression of circRNAs and their linear counterparts in (A) kidney and (B) prostate cancer tissue samples depending on RIN and their quantification as relative quantities and normalized relative quantities. Linear regression line analyses of the expression levels presented as relative quantities (RQs) and normalized relative quantities (NRQs) in Figure 4 were performed. Data were calculated both for samples over the whole range of RIN and only for samples with RIN > 6. Statistical differences between the slopes of RQs and NRQs, and their deviation from zero were assessed. The results have been summarized in the Tables S13 and S14 and have been described in Results.

To counter the argument that the model experiments with thermally degraded RNA samples do not reflect the processes that influence RNA integrity during sample collection, storage, and processing, we analyzed the expression changes in tissue samples with varying RIN values due to sampling conditions. We used RNA isolated from kidney cancer samples and assessed the expression levels of the previously mentioned circRNAs and their linear counterparts, including those of the reference genes *PPIA* and *TBP*. In addition, we used prostate cancer samples and included *circCSNK1G3.* A circRNA deregulated in PCa 38 and the established mRNA normalizers *ALAS1* and *HPRT1* for PCa expression studies 44 in our assay panel. As RIN values between 5 to 7 have been recommended as suitable integrity criteria 17-19, 21, we used the RIN value of 6 as cutoff to obtain two groups with approximately similar number of patients. According to the sample size calculation described in Materials and Methods, we analyzed 61 ccRCC tissue samples, 28 with RIN ≤ 6 and 33 with RIN > 6, and 57 PCa tissue samples, 26 with RIN ≤ 6 and 31 with RIN > 6 (Figure 4). The clinicopathological characteristics of the two RIN-related patient groups (Tables S2 and S3) did not differ significantly (*P-*values from 0.150 to 1.000; except for the age of patients with PCa). Thus, the contribution of the clinicopathological factors to possible expression differences in the two RIN groups may be ignored. Expression of all circRNAs and mRNAs calculated as relative quantities in both kidney and prostate cancer patients were significantly lower in RNA samples with RIN values ≤ 6 than in those with RIN values > 6 (Figure 4A-B). In contrast, using the normalization approach with two conventional reference genes for the respective cancer type, the expression of most circRNAs and their linear counterparts were found not to differ between the two RIN groups in both cancers, except *circEGLN3* and *linEGLN3,* which were found to differ in ccRCC samples even after normalization (Figure 4A-B). Even if RIN 7 was selected as cutoff, the differences remained (Table S12). The normalization of all circRNAs and linear transcripts (NRQ) did however result in significantly lower slopes in the linear regression analysis in contrast to the slopes obtained when analyzing the relative quantities (RQ) (Figure 5 with detailed statistics in Tables S13 and S14). Furthermore, none of the slopes of measured RNA differed significantly from zero when analyzing only the ccRCC samples with RIN values > 6 (Figure 5; *P*-values from 0.169 to 0.771 with detailed statistics in the Table S13). In prostate cancer samples with RIN > 6, the slopes of the linear regression equations of all normalized RNAs did not differ from zero, but differed significantly when the relative quantities of *linCSNK1G3, circRHOBTB3*, and *linRHOBTB3* were analyzed (Table S14). Collectively, the adverse effect of RIN can be only partially compensated by adjusting the expression data of the target RNAs to those of suitable reference genes, although this was dependent on the RNA variables, tissue source, reference genes, and RIN of the RNA samples. As explained above at the example of the model experiment, miscorrection may specifically occur when reference genes and the target RNAs have different degradation patterns.

## Discussion

It is well acknowledged that the integrity of the starting RNA material is one of the decisive factors for obtaining reliable gene expression data using RT-qPCR. It is therefore of particular practical importance to analyze the adverse effects of RNA degradation on circRNA expression levels in clinical tissue samples. This concerns both the RNA samples isolated from fresh tissue samples, either immediately frozen or preserved in RNA storage solution, as well as from formalin-fixed paraffin-embedded (FFPE) tissue, the most routinely preserved tissue material for diagnostic purposes 42. The quality of the total RNA isolated from clinical samples may be affected by the delayed processing interval between the acquisition and stabilized storage of samples 48. As a result, the ongoing endogenous RNase activities lead to the degradation of the total RNA. In the case of FFPE samples, the fixation process causes additional degradation. However, RNA integrity may also be compromised by the sample transport, handling of the samples, and the purity of the reagents and tubes used for the RNA isolation or by storage 20. Thus, estimation of the extent of degradation of the starting RNA sample is a fundamental prerequisite for reliable downstream RT-qPCR measurements, which is specified in the MIQE guidelines 42. As briefly stated in the Introduction, the extent of degradation of the starting material can be assessed using the RIN obtained from microfluidics-based RNA analysis (Agilent) or the RNA quality indicator (Bio-Rad), or using different RT-qPCR-based tests such as the 3':5' ratio assay or 5':3' ratio assay, and an assay based on testing of different amplicons 17, 18, 20, 49-52.

In this study, we determined the integrity of all total RNA samples using RIN as the most frequently used integrity indicator of total RNA, which is also comprehensible for a broad scientific community. To focus our investigations on the effect of RNA integrity on expression analysis, other interfering factors of RT-qPCR, such as primer design, cDNA synthesis, and adjustments for PCR efficiency were avoided 18, 45, 46, 53. This was also necessary, as additional comparative analyses of the circRNAs with the corresponding linear transcripts of their host genes had to be accomplished. Therefore, PCR primers were designed for short amplicons < 250 bp independent of RNA integrity 18, 54. A mixture of random and oligo(dT) primers was used for efficient cDNA synthesis for all measurements 53, an additional reverse transcription experiment with separate random hexamer and oligo(dT)_18_ primers was performed for circRNA validation (Figure S3), and PCR efficiency-adjusted expression was calculated using the qbase^+^ algorithm 45, 46. Furthermore, the *in vitro* model experiments were performed with thermally degraded total RNA isolated from two cell lines and a kidney cancer tissue pool. This approach has been applied in other studies 19, 55. Comparative studies on different artificial degradative processes for total RNA have shown that thermal degradation changes RNA integrity in a manner that mimics that of degradation by ubiquitous RNases 49, 52, 56.

After carefully considering these analytical requirements and the acceptable limits of data repeatability and reproducibility, we concluded that the integrity of total RNA samples significantly affects the accuracy of the RT-qPCR read-outs for circRNA expression analysis. To the best of our knowledge, this is the first systematic study on the effect of RNA integrity on RT-qPCR analysis of circRNAs compared to that of mRNAs. The results of the model studies on artificially degraded total RNA samples (Figures 2A-C and 3A-C) and those on clinical samples with different RIN values (Figures 4A, B and 5A, B) indicated that circRNA expression analysis is affected in a manner similar to those of mRNAs in degraded RNA samples. This clearly contrasts the robustness of miRNA expression (*let-7a-5p* and *miR-17-5p*) under adverse RNA integrity conditions shown in this study (Figure 2), which is in agreement with previous observations 17, 22. Apart from these general observations regarding the effect of RNA integrity, difference in expression was observed between individual circRNAs (for example, *circEGLN3* vs. *circRHOBTB3*; Figure 2A) and between circRNAs and their corresponding linear transcripts (for example, *circRHOBTB3* vs. *linRHOBTB3*, Figure 2C). The intracellular stability of circRNAs was considered advantageous for their use as biomarkers 1, 7, 24-26. However, the total RNA undergoes degradation by different endonucleases as soon as a tissue is removed from the body, which is active at different pH values and prefer different substrates 57. As a result, the expression of circRNAs is altered in a manner similar to that of mRNAs if the sample is not stabilized immediately. An overview of 25 randomly selected circRNA tissue expression studies in different cancers published between 2015 and 2020 (Table S1) revealed that the RNA integrity was mostly not reported and has not been considered so far as possible adverse effect on circRNA measurements. However, per MIQE guidelines 42, this is the basic requirement for generating robust data for biomarker studies and clinical decision-making in future.

Furthermore, these results indicated that the evaluation of circRNA expression in clinical tissue samples is associated with the same problems as with the measurement of mRNA expression: (a) can measurements of circRNAs in degraded RNA samples be corrected to the (probable) initial value and (b) is there a critical limit of RNA integrity that should not be crossed for obtaining reliable results? As circRNA and mRNA expression decrease similarly with reduction in RIN (Figure 2A-C), the percentage changes from the initial value are significantly smaller when mRNA expression is used for normalization, which was *TBP* and *PPIA* in our model study (Figure 3). This approach corresponds to the procedure that is now generally used for the normalization of mRNA in RNA samples degraded via different ways 17-19, 23. The more is the similarity in the degradation profiles of target and reference RNAs, the smaller is the deviation of the corrected value of the degraded sample from the expected value of the intact sample 23. An alternative, particularly for practical reasons, is the combination of several reference genes with different degradation profiles into a RIN-based algorithm that should be used for all circRNA measurements in a study. For example, Cheung et al. 19 developed a special RIN-based corrective algorithm with nine reference genes for estimating mRNA levels in degraded RNA samples. Furthermore, RIN 5-7 was recommended as the lower limit for obtaining reliable mRNA and miRNA expression data using RT-qPCR 17, 18, 58, 59. However, from a practical point of view, it is advisable to combine the correction-based and limit-based assessment, as has been done in this study. This is also highlighted by the finding that even the normalization was not able to sufficiently harmonize the apparent differences of *circEGLN3* and *linEGLN3* in ccRCC samples. Thus, based on our results (Figures 4 and 5), we would suggest a RIN value of six as the limit and the use of at least two specific reference genes, *PPIA* and *TBP*, for kidney cancer samples or *ALAS1* and *HPRT1* for prostate cancer samples 43, 44. Under these conditions, satisfactorily bias-corrected circRNA expression data can be obtained in the model experiments (Figure 3) and for our clinical samples (Figures 4 and 5). We believe that this approach is suitable for obtaining meaningful circRNA expression data for a "fit-for-purpose" procedure applicable in future clinical studies 60.

As already emphasized above, it is not surprising that the generally accepted procedures for normalizing RT-qPCR measurements have received little attention in circRNA expression studies so far 17, 18, 42, 45, 46. For example, the "obsolete" housekeeping genes *ACTB* and *GAPDH* are used frequently as single normalizers (Table S1). Furthermore, normalization with at least two validated reference genes, a long-established approach in mRNA expression studies 47, is rarely applied (Table S1). In this respect, improvements should also be made for future circRNA studies. Zhong et al. 61 recently recommended the use of *hsa_circ_0000284* and *hsa_circ_0000471* as generally applicable reference genes in all circRNA expression studies. The authors only examined the stability of circRNAs with regard to their resistance to RNase R digestion. However, the suitability of these circRNAs as stable expressed normalizers in expression studies and also their degradation pattern in relation to the RNA integrity need further detailed investigations.

Our study has some limitations. First, only few circRNAs and only two carcinoma types were analyzed. However, the general and differential degradation behavior of individual circRNAs, and their dependence on the tissue under investigation are already evident. In addition, the strict adherence of the investigation to the MIQE guidelines, the robustness of the analytical performance data, the calculated sample size (α = 0.05; β = 0.10) that excludes type I and II errors as far as possible, and the comparable clinicopathological characteristics of the two different RIN groups support the general validity of the measured and evaluated data. Second, our results of circRNA measurements refer only to the RT-qPCR methodology and did not consider the particularities of other analytical techniques like microarray, hybridization methods, RNAseq, and next generation sequencing. Irrespective of the situation with other methods, it is necessary to consider this issue for RT-qPCR measurements of clinical samples. Moreover, the specific problem of FFPE material needs clarification 31, 62-64. New RNA quality metrics, which are more sensitive than the RIN values generally used up to now, are recommended to define the preanalytical RNA conditions for reliable expression analyses in future studies 63, 64. These are, for example, the DV 200 that represents the percentage of RNA fragments longer than 200 nucleotides or the Q-score that characterizes the ratio of the *GAPDH* amplicons of 165 bp to 80 bp. A multiphase development process is necessary for the introduction of new circRNA-based assays into clinical practice 12. After the identification and validation of a circRNA, clinical validation must be performed. This requires robust assays of RT-qPCR measurements. All decisive parameters of the pre-analytical phase (sample collection, processing, storage conditions), the analytical phase (RNA isolation protocols, analytical inclusion/exclusion criteria of samples for further analysis, quantification principles with performance data) and the post-analytical phase (data evaluation and normalization approach) must be defined.

In summary, this is the first systematic study comparing the stability of circRNAs with those of their linear mRNA transcripts from the same host gene in clinical tissue samples. CircRNAs showed similar degradation profiles as mRNAs in isolated total RNA samples. The degradation behavior differed between individual circRNAs and was tissue-specific. Based on RNA integrity as the indicator of RNA degradation, the RT-qPCR read-outs of circRNAs were found to be affected similar to those of mRNAs. This has not been sufficiently considered in previous studies on circRNA expression. Based on the observations of RT-qPCR analysis of mRNAs, we concluded that the adverse RNA integrity effect can be partly compensated in an appropriate manner if (a) tissue-specific reference genes are used as normalizers that are validated and recommended in their number by normalization software like geNorm in the software package qbase^+^ (corrective-based approach) 45, 46 and (b) if only RNA samples within a certain integrity limit (limit-based approach; in the present study: RIN > 6) are selected as appropriate study samples. Such a combined approach, adapted always on the objectives of the respective study, allows the exclusion of pre-analytically unsuitable samples and provides measurement results applicable for clinical practice. This is the prerequisite for exploiting the full potential of circRNAs as diagnostic, prognostic, and predictive biomarkers in future circRNA tissue expression studies 12.

## Supplementary Material

Supplementary figures and tables.

## Figures and Tables

**Table 1:** List of circRNAs and their linear mRNA counterparts in this study

RNA name in the manuscript ^a^	References in circBase ^b^ or NCBI Genbank ^c^	Official gene symbol of the host gene and its full name
*circEGLN3*	hsa_circ_0101692	*EGLN3*, egl-9 family hypoxia inducible factor 3
*linEGLN3*	NM_022073.4	
*circRHOBTB3*	hsa_circ_0007444	*RHOBTB3*, Rho related BTB domain containing 3
*linRHOBTB3*	NM_014899.4	
*circCSNK1G3*	hsa_circ_0001522	*CSNK1G3*, casein kinase 1 gamma 3
*linCSNK1G3*	NM_001044723.2	
*circRNA4* ^d^	hsa_circ_0001900	*CAMSAP1*, calmodulin regulated spectrin associated protein 1
*circRNA9* ^d^	hsa_circ_0001423	*AFF1*, AF4/FMR2 family member 1

a: In the text, the abbreviated names of the circRNAs (circ+host gene symbol) and mRNAs (lin+host gene symbol) are used to facilitate the readability of the text. b: Database circBase, http://www.circbase.org 36. c: Genbank of National Center for Biotechnology Information, https://www.ncbi.nlm.nih.gov/. d: According to Memczak et al 4.

## Abbreviations

%RSD: percentage relative standard deviation
ACTB: actin, beta
ALAS1: 5'‑aminolevulinate synthase 1
ccRCC: clear cell renal cell carcinoma
circ+HOST GENE SYMBOL: circular ribonucleic acid derived from the host gene
circRNA: circular ribonucleic acid
Cq: quantification cycle
CSNK1G3: casein kinase 1 gamma 3
EGLN3: egl-9 family hypoxia inducible factor 3
FFPE: formalin-fixed paraffin-embedded
GAPDH: glyceraldehyde-3-phosphate dehydrogenase
HPRT1: hypoxanthine phosphoribosyltransferase 1
lin+HOST GENE SYMBOL: linear messenger ribonucleic acid derived from the host gene
MIQE: minimum information for publication of quantitative real-time PCR
miRNA: microribonucleic acid
mRNA: messenger ribonucleic acid
NRQ: normalized relative quantity
PCa: prostate cancer
PPIA: peptidylprolyl isomerase A
RHOBTB3: rho related BTB domain containing 3
RIN: ribonucleic acid integrity number
RQ: relative quantity
RT-qPCR: quantitative real-time reverse-transcription polymerase chain reaction
SD: standard deviation
TBP: tata-box binding protein
