# Supplementary material for: Instability of circular RNAs in clinical tissue samples impairs their reliable expression analysis using RT-qPCR: from the myth of their advantage as biomarkers to reality
Source: Theranostics. 2020 Jul 23;10(20):9268–79. doi: 10.7150/thno.46341 (PMC7415809; doi:10.7150/thno.46341)
Supplement: Supplementary file 1 — Supplementary figures and tables. [file thnov10p9268s1.pdf]

Supplementary Material

Instability of circular RNAs in clinical tissue samples impairs their reliable expression analysis using RT-qPCR: from the myth of their advantage as biomarkers to reality

Hannah Rochow, Antonia Franz, Monika Jung, Sabine Weickmann, Bernhard Ralla, Ergin Kilic, Carsten Stephan, Annika Fendler, Klaus Jung

Inhaltsverzeichnis

1. Pre-analytical and analytical characteristics of published circRNA tissue expression studies in cancers - 2 -
Table S1 - 2 -

2. Clinicopathological characteristics of the own study cohorts - 4 -
Table S2. Renal cell carcinoma patients - 4 -
Table S3. Prostate cancer patients - 5 -

3. RT-qPCR methodology - 6 -
Table S4. MIQE checklist - 6 -
Figure S1. Bioanalyzer 2100 results of RNA samples after heat degradation - 10 -
3.1. cDNA synthesis - 11 -
3.1.1. cDNA synthesis of circRNAs and mRNAs - 11 -
Table S5A. cDNA synthesis using the Maxima First Strand cDNA Synthesis Kit. - 11 -
Table S5B. cDNA synthesis using the Transcriptor First Strand cDNA Synthesis Kit. - 11 -
3.1.2. cDNA synthesis for microRNAs - 11 -
3.2. qPCR- measurements - 11 -
3.2.1. Quantification of circRNAs and mRNAs - 11 -
Table S6. List of backsplice junctions of circRNAs and their primers - 13 -
Table S7. QPCR target information of the linear counterparts of the circRNAs and of the reference genes for normalization - 14 -
Table S8. List of primers - 15 -
Protocols for all LightCycler runs in SYBR Green and Probe assay format - 16 -
3.2.2. Quantification of miRNAs - 18 -
Table S9. TaqMan MicroRNA Assays - 18 -
3.3. Performance data of the assays - 18 -
Figure S2. Amplicon analyses of circCSNK1G3, circEGLN3, and circRHOBTB3 - 19 -
Figure S3. Random vs. oligo(dT) primers for cDNA synthesis of circRNAs - 20 -
Table S10. Characteristics of the PCR standard curves - 21 -
Table S11. Repeatability and reproducibility of RT-qPCR measurements. - 22 -

4. Statistical data regarding the relationship of expression results to RIN values - 23 -
Table S12. *P*-values of the expression data calculated as relative quantities and normalized quantities using RIN 6 and 7 - 23 -
Table S13. Regression line analysis of circRNAs and their linear counterparts in kidney cancer (Figure 5A) - 24 -
Table S14. Regression line analysis of circRNAs and their linear counterparts in prostate cancer (Figure 5B) - 25 -

5. References - 25 -

1. Pre-analytical and analytical characteristics of published circRNA tissue expression studies in cancers

Supplemental Table S1. Analytical characteristics of 25 randomly selected studies of circRNA expression in different cancers between 2015 and 2020 [a]

No.	Reference, Year	Cancer type	Tissue collection and storage	Analysis of RNA integrity	Applied references for normalization
1	Li et al., 2015 [1]	Gastric cancer	5	No	GAPDH
2	Ahmed et al., 2016 [2]	Ovarian cancer	1	No	ACTB, GAPDH
3	Lu et al., 2017 [3]	Breast cancer	5	Standard denaturing gel electrophoresis, no further comments	GAPDH
4	Zhu et al., 2017 [4]	Lung adenocarcinoma	1	No	GAPDH
5	Dang et al., 2017 [5]	Gastric cancer	5	Denaturing gel electrophoresis, no further comments	GAPDH
6	Weng et al., 2017 [6]	Colorectal cancer	1	No	U6, GAPDH
7	Cao et al., 2017 [7]	Hypopharyngeal cancer	4	Denaturing gel electrophoresis, no further comments	ACTB
8	Chen et al, 2017 [8]	Gastric cancer	5	No	GAPDH
9	Huang et al, 2017 [9]	Hepatocellular cancer	4	No	GAPDH
10	Zhang et al., 2018 [10]	Lung adenocarcinoma	1	No	ACTB
11	Sun et al., 2018 [11]	Oral squamous cancer	2	Denaturing gel electrophoresis, no comments	ACTB
12	Huang et al., 2019 [12]	Kidney cancer	4	No	GAPDH
13	Chen et al., 2019 [13]	Glioma	0	No	GAPDH
14	Lu et al., 2020 [14]	Colon cancer	4	No	GAPDH
15	Jin et al., 2020 [15]	Melanoma	3	No	GAPDH
16	Xing et al., 2020 [16]	Esophageal cancer	2	No	Only Cq based
17	Sun et al., 2020 [17]	Thyroid cancer	2	No	GAPDH
18	Liu et al., 2020 [18]	Pancreatic cancer	3	No	GAPDH
19	Zhang et al., 2020 [19]	Laryngeal carcinoma	3	No	ACTB
20	Li et al, 2020 [20]	Osteosarcoma	0	No	GAPDH

No.	Reference, Year	Cancer type	Tissue collection and storage	Analysis of RNA integrity	Applied references for normalization
21	Zhou et al., 2020 [21]	Multiple myeloma	0	Agilent analysis, no further comments	GAPDH, after RNA treatment with RNase R
22	Kong et al., 2020 [22]	Prostate cancer	0	No	ACTB
23	Meng et al., 2020 [23]	Cervical cancer	0	No	GAPDH
24	Wei et al., 2020 [24]	Hepatocellular cancer	0	No	GAPDH
25	Yu et al., 2020 [25]	Bladder cancer	0	No	GAPDH

[a] The studies were reviewed only with regard to the data of tissue collection and storage, RNA integrity, and reference standards for normalization.
[b] Information on tissue collection and storage in the studies was categorized with following specifications: 0=no detailed information regarding collection and storage; 1=fresh frozen; 2=stored at -80 °C; 3=collected and stored in liquid nitrogen until use; 4=collected in liquid nitrogen and stored at -80 °C until use; 5=collected in RNA storage solution and stored at -80 °C until use.

ACTB: actin beta; GAPDH: glyceraldehyde-3-phosphate dehydrogenase; U6: RNA, U6 small nuclear 1.

2. Clinicopathological characteristics of the study own cohorts in relation to the degraded RNA samples

Supplemental Table S2. Clinicopathological characteristics of patients suffering from clear cell renal cell carcinoma.

Characteristics	Total	Patient samples with RIN ≤6	Patient samples with RIN >6	*P* value [a]
Patients, no. (%)	61 (100)	28 (46)	33 (54)	
Sex, female/male; no. (%)	20/41 (33/67)	8/20 (29/71)	12/21 (36/64)	0.591
Age, years, median (IQR)	61 (56-69)	60 (57–66)	64 (56–70)	0.311
Pathological stage, no. (%)				
pT1a	8 (13)	3 (11)	5 (15)	0.329
pT1b	14 (23)	6 (21)	8 (24)	
pT2	5 (8)	1 (4)	4 (12)	
pT3a	22 (36)	13 (46)	9 (27)	
pT3b	8 (13)	4 (14)	4 (12)	
pT3c	3 (5)	0	3 (9)	
unclassified	1 (2)	1 (4)	0 (0)	
Metastatic status, no. (%)				
negative	47 (77)	20 (72)	27 (82)	1.000
positive	10 (16)	4 (14)	6 (18)	
unclassified	4 (7)	4 (14)	0 (0)	
TNM stage grouping, no. (%) [b]				
I	22 (36)	9 (32)	13 (39)	0.361
II	5 (8)	1 (4)	4 (12)	
III	24 (39)	14 (50)	10 (30)	
IV	10 (16)	4 (4)	6 (18)	
Tumor size, mm, median (IQR)	65 (49-87)	75 (60-118)	60 (47-81)	0.309
Surgical margin, no. (%)				
negative	47 (77)	19 (68)	28 (85)	0.336
positive	12 (20)	7 (25)	5 (15)	
unclassified	2 (3)	2 (7)		
Fuhrman grade, no. (%)				
G1	4 (7)	1 (4)	3 (9)	0.689
G2	31 (51)	13 (46)	18 (55)	
G3	17 (28)	6 (21)	11 (33)	
G4	3 (5)	2 (7)	1 (3)	
unclassified	6 (9)	6 (21)	0 (0)	

[a] Calculated with Fisher's exact test, Chi-squared test or Mann-Whitney *U* test between the two RIN groups without considering unclassified data.
[b] TNM stage grouping according to UICC classification system.
G: histopathological grading according to Fuhrman; IQR: interquartile range; pT: pathological tumor classification; RIN: RNA integrity number.

Supplemental Table S3. Clinicopathological characteristics of patients suffering from prostate cancer.

	Total	Patient samples with RIN ≤6	Patient samples with RIN >6	*P* value[a]
Patients, no. (%)	57 (100)	26 (46)	31 (54)	
Age, years, median (IQR)	67 (62–71)	71 (66–73)	65 (61–69)	0.004
PSA, µg/L, median (IQR)	8.3 (5.95–14.0)	11.4 (6.7–17.8)	7.4 (5.7–12.2)	0.157
Prostate volume, cm^3, median (IQR)	31 (25–38)	32 (28–36)	30 (25–38)	0.539
DRE, no. (%)				
non-suspicious	30 (53)	13 (50)	17 (55)	1.000
suspicious	16 (28)	7 (27)	9 (29)	
unclassified	11 (19)	6 (23)	5 (16)	
pT status, no. (%)				
pT2a	1 (2)	1 (4)	0 (0)	0.726
pT2c	22 (39)	10 (38)	12 (39)	
pT3a	14 (25)	6 (23)	8 (26)	
pT3b	19 (33)	8 (31)	11 (35)	
unclassified	1 (2)	1 (4)	0 (0)	
ISUP Grade groups, no. (%)				
1	8 (14)	2 (7.5)	6 (19)	0.150
2	19 (33)	9 (35)	10 (32)	
3	15 (26)	4 (15)	11 (35)	
4	5 (9)	3 (12)	2 (7)	
5	8 (14)	6 (23)	2 (7)	
unclassified	2 (4)	2 (7.5)	0 (0)	
pN status, no. (%)				
pN0/Nx	51 (89)	22 (85)	29 (94)	0.396
pN1	6 (11)	4(15)	2 (6)	
Surgical margin, no. (%)				
negative	28 (49)	12 (46)	16 (52)	1.000
positive	28 (49)	13 (50)	15 (48)	
unclassified	1 (2)	1 (0)	0 (0)	

[a] Calculated with Fisher's exact test, Chi-squared test or Mann-Whitney *U* test between the two RIN groups without considering unclassified data.
DRE: digital rectal examination; IQR: interquartile range; ISUP: histopathological grade system based on Gleason score according to the International Society of Urologic Pathology; pN: lymph node status; PSA: total prostate specific antigen; pT: pathological tumor classification; RIN: RNA integrity number.

3. RT-qPCR methodology

Supplemental Table S4. MIQE checklist according to Bustin et al. [26].

ITEM TO CHECK	IMPORTANCE	CHECKLIST	WHERE; COMMENT
**EXPERIMENTAL DESIGN**			
Definition of experimental and control groups	E	Yes	Main text: Materials and Methods; Tables S2 and S3
Number within each group	E	Yes	Main text: Materials and Methods; Results; Tables S2 and S3
Assay carried out by core lab or investigator's lab?	D	Yes	Investigator's lab
Acknowledgement of authors' contributions	D	Yes	Section Acknowledgements
**SAMPLE**			
Description	E	Yes	Main text: Results; Materials and Methods.
Volume/mass of sample processed	D	Yes	Main text: Results; Materials and Methods
Microdissection or macrodissection	E	Yes	Main text: Materials and Methods
Processing procedure	E	Yes	Main text: Materials and Methods
If frozen - how and how quickly?	E	Yes	Main text: Materials and Methods
If fixed - with what, how quickly?	E	Yes	Main text: Materials and Methods
Sample storage conditions and duration (esp. for FFPE samples)	E	Yes	Main text: Materials and Methods
**NUCLEIC ACID EXTRACTION**			
Procedure and/or instrumentation	E	Yes	Main text: Materials and Methods
Name of kit and details of any modifications	E	Yes	Main text: Materials and Methods
Source of additional reagents used	D	N/A	Not used
Details of DNase or RNase treatment	E	Yes	Main text: Materials and Methods: RNA extraction, on-column DNase digestion
Contamination assessment (DNA or RNA)	E	Yes	Main text: Methods and Materials. Supplementary Material: Genomic DNA contamination was excluded by control experiments without reverse transcription of RNA for all target genes
Nucleic acid quantification	E	Yes	Main text: Materials and Methods, spectrophotometric
Instrument and method	E	Yes	Main text: Materials and Methods, Nanodrop
Purity (A260/A280)	D	Yes	Main text: Materials and Methods
Yield	D	Yes	Main text: Materials and Methods
RNA integrity method/instrument	E	Yes	Main text: Materials and Methods: RIN; Bioanalyzer 2100, Agilent RNA 6000 Nano Chip Kit
RIN/RQI or Cq of 3' and 5' transcripts	E	Yes	Main text: Materials and Methods: RIN; Bioanalyzer 2100, Agilent; see Figure S1
Electrophoresis traces	D	Yes	see RNA integrity: Agilent electrophoresis
Inhibition testing (Cq dilutions, spike or other)	E	Yes	Supplementary Material: Cq dilution, see standard curve characteristics in Supplemental Table S10
**REVERSE TRANSCRIPTION**			
Complete reaction conditions	E	Yes	Main text: Materials and Methods; Supplementary Material: RT-qPCR methodology, cDNA synthesis in 3.1.1
Amount of RNA and reaction volume	E	Yes	Main text: Materials and Methods; Supplementary Material: RT-qPCR methodology, cDNA synthesis in 3.1.1

Priming oligonucleotide (if using GSP) and concentration	E	Yes	Main text: Materials and Methods; Supplementary Material: RT-qPCR methodology, cDNA synthesis in 3.1.1
Reverse transcriptase and concentration	E	Yes	Main text: Materials and Methods; Supplementary Material: RT-qPCR methodology, cDNA synthesis in 3.1.1
Temperature and time	E	Yes	Main text: Materials and Methods; Supplementary Material: RT-qPCR methodology, cDNA synthesis in 3.1.1
Manufacturer of reagents and catalogue numbers	D	Yes	Main text: Materials and Methods; Supplementary Material: RT-qPCR methodology, cDNA synthesis in 3.1.1.
Cqs with and without RT	D	Yes	Supplementary Material: RT-qPCR methodology: neg. results; see also comment on DNase treatment. There were no Cqs <40 in reactions without RT
Storage conditions of cDNA	D	Yes	Main text. Materials and Methods; storage at -20 °C
**qPCR TARGET INFORMATION**			
Gene symbols	E	Yes	Main text: Table 1; Supplementary Material: Table S6–S8
If multiplex, efficiency and LOD of each assay.	E	N/A	Only singleplex qPCR
Sequence accession number	E	Yes	Main text: Table 1; Supplementary Material: Supplemental Table S6–S9.
Location of amplicon	D	Yes	Supplementary Material: Supplemental Tables S6-S7
Amplicon length	E	Yes	Supplementary Material: Supplemental Tables S6-S7; Bioanalyzer 2100 DNA1000 expert series II Chip analysis: Figure S2
In silico specificity screen (BLAST, etc)	E	Yes	Main text: Materials and Methods; Supplementary Material: RT-qPCR methodology. All primers and amplicons were checked by screens in different databases, see URL links in 3.2.1
Pseudogenes, retropseudogenes or other homologs?	D	N/A	
Sequence alignment	D	Yes	Supplementary Material: RT-qPCR methodology, see URL links as mentioned above in 3.2.1. Using NCBI-based Megablast against standard Nucleotide collection databases (nr/nt) and RefSeq, filtered Homo sapiens (taxid.9606). Analyses using databases circBase and CircInteractome
Secondary structure analysis of amplicon	D	No	
Location of each primer by exon or intron (if applicable)	E	Yes	Supplementary Material: Supplemental Tables S6-S7. Analysis of different databases: Ensembl NCBI nucleotide, circBAse and CircInteractome
What splice variants are targeted?	E	N/A	
**qPCR OLIGONUCLEOTIDES**			
Primer sequences	E	Yes	Supplementary Material: RT-qPCR methodology with Supplemental Tables S8
RTPrimerDB Identification Number	D	N/A	
Probe sequences	D	Yes	Supplementary Material: RT-qPCR methodology, UPL probes only for ALAS1 and HPRT1 in 3.2.1
Location and identity of any modifications	E	N/A	No modifications
Manufacturer of oligonucleotides	D	Yes	TIB MolBiol (Berlin, Germany); Applied Biosystems; Probes from Roche
Purification method	D	Yes	TIB MolBiol: GSF purification

**qPCR PROTOCOL**			
Complete reaction conditions	E	Yes	Main text: Materials and Methods. Supplementary Material: RT-qPCR methodology
Reaction volume and amount of cDNA/DNA	E	Yes	Main text: Materials and Methods. Supplementary Material: RT-qPCR methodology
Primer, (probe), Mg++ and dNTP concentrations	E	Yes	Main text: Materials and Methods. Supplementary Material: RT-qPCR methodology
Polymerase identity and concentration	E	Yes	Main text: Materials and Methods. Supplementary Material: RT-qPCR methodology
Buffer/kit identity and manufacturer	E	Yes	Main text: Materials and Methods. Supplementary Material: RT-qPCR methodology
Exact chemical constitution of the buffer	D	No	The manufacturer does not provide this information
Additives (SYBR Green I, DMSO, etc.)	E	Yes	Main text: Materials and Methods. Supplementary Material: RT-qPCR methodology, SYBR Green in ready-to-use soft master
Manufacturer of plates/tubes and catalogue number	D	Yes	Supplementary Material: RT-qPCR methodology in 3.1.1 and 3.2.1
Complete thermocycling parameters	E	Yes	Main text: Materials and Methods. Supplementary Material: RT-qPCR methodology for all runs in 3.2.1
Reaction setup (manual/robotic)	D	Yes	Manual setup
Manufacturer of qPCR instrument	E	Yes	Main text: Materials and Methods: LightCycler 480 (Roche)
**qPCR VALIDATION**			
Evidence of optimisation	D	Yes	Supplementary Material: RT-qPCR methodology: all run conditions of qPCRs were optimized, for circEGLN3, linEGLN3, circRHOBTB3, and linRHOBTB3 see also ref. [27]; for reference genes [28, 29] with primers as indicated in Table S8; for circCSNK1G3 see Supplemental Figure S2 according to Chen et al. [30].
Specificity (gel, sequence, melt, or digest)	E	Yes	Supplementary Material: RT-qPCR methodology with Supplemental Figure S2 for circEGLN3, circRHOBTB3, and circCSNK1G3 (Agilent electropherogram, melting curves on LightCycler); other circRNAs also in ref. [27])
For SYBR Green I, Cq of the NTC	E	Yes	Main text: Materials and Methods; Supplementary Material: RT-qPCR methodology, no Cqs <40 in reaction without RT
Standard curves with slope and y-intercept	E	Yes	Supplementary Material: RT-qPCR methodology; 3.3. Performance data, Table S10
PCR efficiency calculated from slope	E	Yes	Supplementary Material: RT-qPCR methodology; 3.3. Performance data, Table S10
Confidence interval for PCR efficiency or standard error	D	Yes	Supplementary Material: RT-qPCR methodology; 3.3. Performance data, Table S10
r2 of standard curve	E	N/A	Not provided by the LC480 software
Linear dynamic range	E	Yes	Supplementary Material: RT-qPCR methodology; 3.3. Performance data, Table S10 with endpoints of standard curves
Cq variation at lower limit	E	Yes	Supplementary Material: RT-qPCR methodology, Supplemental Table S10 with Cq range of the measured samples, only two samples (see Table S10) with outside of the dynamic range

Confidence intervals throughout range	D	N/A	
Evidence for limit of detection	E	Yes	Supplementary Material: RT-qPCR methodology, Supplemental Table S10: samples with Cq values in the dynamic range of the standard curves except two samples (see Table S10)
If multiplex, efficiency and LOD of each assay.	E	N/A	No multiplex assays
**DATA ANALYSIS**			
qPCR analysis program (source, version)	E	Yes	Main text: Materials and Methods. Supplementary Material: RT-PCR methodology. 3.3. Performance data with Supplemental Table S10 (LightCycler 480 software, release 1.5.1.62 using the "second derivative maximum" method); qbase$^+$ software, version 3.2 (Biogazelle, Zwijnaarde, Belgium) for generation of relative quantities (RQs) and normalized relative quantities (NRQs)
Cq method determination	E	Yes	
Outlier identification and disposition	E	N/A	2 samples with Cqs outside of the standard curve were included in the study
Results of NTCs	E	Yes	Main text: Materials and Methods; Supplementary Material: RT-PCR methodology, no Cqs <40 in reaction without RT
Justification of number and choice of reference genes	E	Yes	Main text: Materials and Methods; Results. Supplementary Material: RT-PCR methodology with reference genes PPIA and TBP according to [28] and ALAS1 and HPRT1 according to [29]
Description of normalisation method	E	Yes	Main text: Materials and Methods; Supplementary Material: RT-PCR methodology: cancer-specific reference genes PPIA and TBP for kidney cancer, ALAS1 and HPRT1 for prostate cancer, see previous comment. Use of the software qbase$^+$
Number and concordance of biological replicates	D	Yes	Concordance is reflected by the performance data given in the repeatability and reproducibility data in Table S11
Number and stage (RT or qPCR) of technical replicates	E	Yes	Main text: Materials and Methods; Supplementary Material: RT-PCR methodology, at least technical duplicates in qPCR
Repeatability (intra-assay variation)	E	Yes	Main text: Materials and Methods; Supplementary Material: RT-PCR methodology, 3.3. Performance data, Table S11
Reproducibility (inter-assay variation, %CV)	D	Yes	Main text: Materials and Methods; Supplementary Material: RT-PCR methodology, 3.3. Performance data, Table S11
Power analysis	D	Yes	Main text: Materials and Methods: Data analysis
Statistical methods for result significance	E	Yes	Main text: Materials and Methods: Data analysis. Results, figures, and figure legends
Software (source, version)	E	Yes	Main text: Materials and Methods: Data analysis, statistical analysis
Cq or raw data submission using RDML	D	No	

E: essential information; D: desirable information if available; N/A: not applicable.

1. A-498 cell: RNA degradation

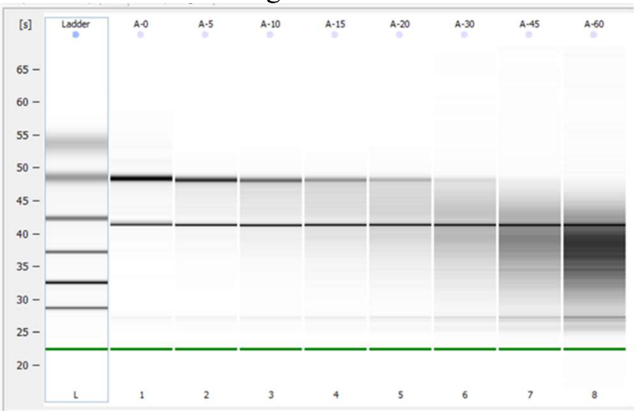

2. HEK-293 cell: RNA degradation

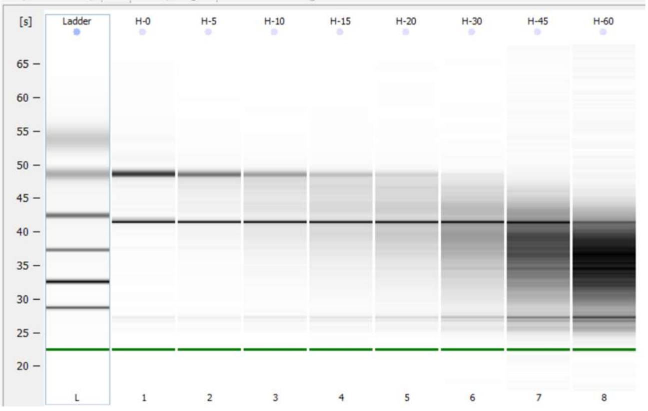

3. Renal tissue pool RNA: RNA degradation

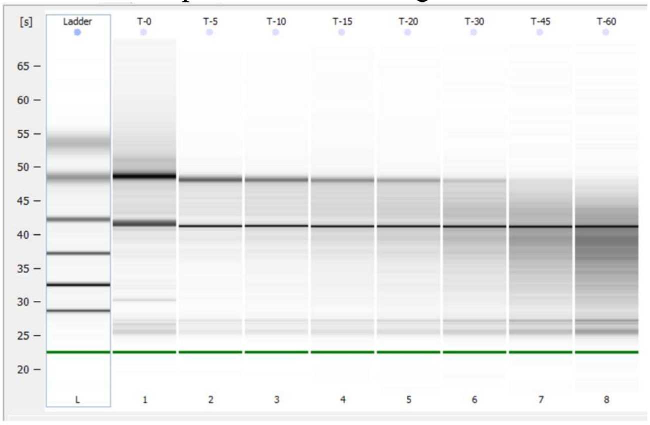

Supplemental Figure S1. Bioanalyzer 2100 results of RNA samples after heat incubation at 80 °C. The time-dependent RIN decay of samples is shown in Figure 1 of the main text.

3.1. cDNA synthesis

3.1.1. cDNA synthesis of circRNAs and mRNAs

Maxima First Strand cDNA Synthesis Kit for RT-qPCR (Thermo Scientific, Waltham, MA, USA; Cat.No. K1642) was used in final reaction volume of 20 µL according to the following protocol:

Supplemental Table S5A. cDNA synthesis using the Maxima First Strand cDNA Synthesis Kit.

Volume (µL)	Reagent/Sample	Components
4	5X Reaction Mix	Reaction buffer, dNTPs, oligo(dT)$_{18,}$ and random hexamer primers without specified concentration
2	Maxima Enzyme Mix	Maxima Reverse Transcriptase (M-MuLV RT) and Thermo Scientific™ RiboLock™ RNase Inhibitor
2	Total RNA (500 ng)	Diluted RNA (gDNA free); see RNA isolation
12	Water, nuclease free	

The RT reaction was carried out in 0.2 mL PCR Soft Tubes (Biozym Scientific GmbH, Germany; Article No. 711080) in a thermal block cycler (Biometra GmbH, Göttingen, Germany) as follows: 10 min at 25 °C, followed by 15 min at 50 °C, and terminated by heating at 85 °C for 5 min; end 4 °C. All cDNA samples were stored at -20 °C until qPCR analysis (see the following Section 3.2.1).

As explained in the Main text: Materials and Methods, cDNA synthesis, we used the Transcriptor First Strand cDNA Synthesis Kit (Life Science Roche, Mannheim, Germany; Cat. No. 04379012001) for the cDNA synthesis of circRNAs for the comparative priming with random hexamer and oligo(dT)$_{18}$ primers according to the following protocol:

Supplemental Table S5B. cDNA synthesis using Transcriptor First Strand cDNA Synthesis Kit.

Volume (µL)	Reagent/Sample	Components and final (1x) concentration
2	Total RNA (500 ng)	1 µg
2<br>or<br>1	Random Hexamer Primer<br>or:<br>Anchored-oligo(dT)$_{18}$ Primer	60 µM<br>or:<br>2.5 µM
9 or 10	Water, PCR Grade	
4	Transcriptor Reverse Transcriptase Reaction Buffer, 5x conc.	50 mM Tris/HCl, 30 mM KCl, 8 mM $MgCl_2$
0.5	Protector RNase Inhibitor	20 U
2	Deoxynucleotide Mix	1 mM each
0.5	Transcriptor Reverse Transcriptase	10 U

The RT reaction conditions were primer dependent. Using random hexamer primers: 10 min at 25 °C, followed by 30 min at 55 °C and inactivation for 5 min at 85 °C; end 4 °C. Using anchored-oligo(dT)$_{18}$ primers, the initial incubation step was omitted, the other temperature steps were identical. Both cDNA samples were stored at -20 °C until qPCR analysis (see the following Section 3.2.1).

3.1.2. cDNA synthesis of microRNAs

The TaqMan MicroRNA Reverse Transcription Kit (Applied Biosystems) was used according to the manufacturer's instructions. Using a Biometra thermal block cycler as mentioned above, the cDNA synthesis was performed in a reaction mixture containing 10 ng of total RNA, 15 nmol of dNTP mix, 50 U AB Multiscribe Reverse Transcriptase, 1x microRNA specific stem-looped RT-primer (AB), 3.75 U AB RNase Inhibitor, and 1x RT buffer. The steps were the following: priming at 16 °C for 30 min, transcription at 42 °C for 30 min, and enzyme inactivation at 85 °C for 5 min. All cDNA samples were stored at -20 °C until qPCR analysis.

3.2. qPCR- measurements

3.2.1. Quantification of circRNAs and mRNAs

All real-time qPCR runs were performed on the LightCycler 480 Instrument (Roche Molecular Systems, Mannheim, Germany) in white 96-well plates (Cat.No. 04729692001) using at least technical

duplicates and resulting mean values were used for further calculations. Maxima SYBR Green qPCR Master Mix (2X) (Thermo Scientific; Cat.No. K0252) was used. The determination of circRNAs is based on the measurement of their specific backsplice junctions using divergent primers [31]. The backsplice junction sequences of the three circRNAs measured in this study and the amplicon characteristics are listed in Supplemental Table S6; information regarding the linear counterparts of the circRNAs and the normalizers are given in Supplemental Table S7.

All primers of circRNAs (divergent) and mRNAs (convergent) were designed using the Primer3 tool (http://bioinfo.ut.ee/primer3/) or with Roche/UPL ProbeFinder web-based software (https://lifescience.roche.com/en_de/brands/universal-probe-library.html#assay-design-center) and are compiled in Supplemental Table S8 [32]. Primers were synthesized by TIB Molbiol GmbH (Berlin, Germany). Expression of mRNAs of peptidylprolyl isomerase (PPIA), TATA-box binding protein (TBP), 5'-aminolevulinate synthase 1 (ALAS1), and hypoxanthine phosphoribosyltransferase 1 (HPRT1) was used for normalization as these genes are generally used as normalizers in expression studies of clear cell renal cell carcinoma and prostate cancer [28, 29]. PPIA was quantified by QuantiTect Primer assay from Qiagen (Supplemental Table S7) in SYBR Green assay format, ALAS1 and HPRT1 were measured with Universal ProbeLibrary-probes (ALAS1 Probe #40 Prod. No: 04687990001; HPRT1 Probe #22 Prod. No: 04686969001) and LightCycler 480 Probes Master from Roche (Prod. No. 04707494001) in hydrolysis probe assay format on the LightCycler 480 (Supplemental Table S8). Quantitative PCR data analysis was done using qbase^{+} software, version 3.2 (Biogazelle, Zwijnaarde, Belgium; www.qbaseplus.com).

In general, genes and primer sequences were checked using the following database links: https://circinteractome.nia.nih.gov/; http://www.circbase.org/; http://www.ensembl.org (Ensembl release 99 - January 2020); https://www.ncbi.nlm.nih.gov/nucleotide; https://blast.ncbi.nlm.nih.gov/Blast.cgi; https://www.ncbi.nlm.nih.gov/tools/primer-blast/.

Supplemental Table S6. List of backsplice junctions of circRNAs and primers used for RT-qPCR detection of circEGLN3, circRHOBTB3, and circCSNK1G3.

**circRNA (circBase ID)**	**Backsplice junction sequence of amplicon**	**Divergent primers**	**Amplicon location (exon no.)**	**Amplicon length (nt)[a]**
circEGLN3<br><br>(hsa_circ_0101692)	**TCCTGCAGACATCCTACTCG**GCCAGCGGTTTACCTGATAGATTCGGCAATGGTGGCTTGCTATCCGGGAAATGGAACAGGTTATGTTCGCCACGTGGACAACCCCAAC**GGTGATGGTCGCTGCATC**	Forward: TCCTGCAGACATCCTACTCG<br>Reverse: GATGCAGCGACCATCACC	Ex5(F)/Ex2(R)	126<br>(129)
circRHOBTB3<br><br>(hsa_circ_0007444)	**TTCTGGGGATGTTTCAAATG**TAATCGAGAAAGTTAAATGCATTTTAAAAACACCAGGAAAGAAAAAATGCCTGTCTTAAAGGCTGAAGCGTCACATTATAACTCTGACTTAAATAACTTG**CTGTTCTGCTGCCAGTGTGT**	Forward: TTCTGGGGATGTTTCAAATG<br>Reverse: ACACACTGGCAGCAGAACAG	Ex7(F)/Ex6(R)	140<br>(138)
circCSNK1G3<br><br>(hsa_circ_0001522)	**GCACCACAGCTACATTTGGA**ATACAGATTCTATAAGCAGTTAGGATCTGGAGCTCTCTATCAATATCAGCTCACATCATTGAAAAGATAATTTTGAAGACATGTTTTGCTGAAAAGACACTAAGAAAAATTTTAC**GAATGGGATGAACATGCTCC**	Forward: GCACCACAGCTACATTTGGA<br>Reverse: GGAGCATGTTCATCCCATTC	Ex3(F)/Ex1(R)	155<br>(149)

[a] Numbers in brackets indicate the lengths of amplicons detected with Agilent Bioanalyzer 2100.

Supplemental Table S7. QPCR target information of the linear counterparts of the circRNAs and the reference genes for normalization.

RNA name in the manuscript	Official gene symbol of the host gene and its full name	NCBI Ref.Seq Accession nos.	Primer location (exon number in RefSeq)	Intron spanning size (nt)	Amplicon size (nt)[a]
linEGLN3	EGLN3, egl-9 family hypoxia inducible factor 3	NM_022073.4	Ex4(F)/Ex5(R)	1037	88 (92)
linRHOBTB3	RHOBTB3, Rho related BTB domain containing 3	NM_014899.4	Ex5(F)/Ex6(R)	3045	94 (96)
linCSNK1G3	CSNK1G3, casein kinase 1 gamma 3	NM_001044723.2	Ex6(F)/Ex8(R)	273/1901	142 (144)
PPIA	PPIA, peptidylprolyl isomerase	NM_021130.5	QuantiTect Primer Assay (Cat.No.: QT-00052311) in Ex 4/5	1412	121 (123)
TBP	TBP, TATA-box binding protein	NM_003194.5	Ex3(F)/Ex5+6 (R)	2311/2285/2602	227 (226)
ALAS1	ALAS1, 5'-aminolevulinate synthase	NM_000688.6	Ex4(F)/Ex5(R)	1128	77 (77)
HPRT1	HPRT1, hypoxanthine phosphoribosyl-transferase 1	NM_000194.3	Ex3(F)/Ex4+5 (R)	11100/3657	126 (119)

[a] Numbers in brackets indicate the lengths of amplicons detected with Agilent Bioanalyzer 2100.

Supplemental Table S8. List of primers.

circRNA (circBase ID)	Divergent Primers	Primer sequences (5'… 3')
circEGLN3 (hsa_circ_0101692)	circEGLN3-F[a]	TCCTGCAGACATCCTACTCG
circEGLN3 (hsa_circ_0101692)	circEGLN3-R[a]	GATGCAGCGACCATCACC
circRHOBTB3 (hsa_circ_0007444)	circRHOBTB3-F[a]	TTCTGGGGATGTTTCAAATG
circRHOBTB3 (hsa_circ_0007444)	circRHOBTB3-R[a]	ACACACTGGCAGCAGAACAG
circCSNK1G3 (hsa_circ_0001522)	circCSNK1G3-F[b]	GCACCACAGCTACATTTGGA
circCSNK1G3 (hsa_circ_0001522)	circCSNK1G3-R[b]	GGAGCATGTTCATCCCATTC
circRNA4 (hsa_circ_0001900)	circRNA4-F[c]	TGTGCTCCTGCTCATACTGGTCAA
circRNA4 (hsa_circ_0001900)	circRNA4-R[c]	TCAGTGCCTCGAAAGAACTTCCGT
circRNA9 (hsa_circ_0001423)	circRNA9-F[c]	GCTCTCCAAAAAGGGGAATC
circRNA9 (hsa_circ_0001423)	circRNA9-R[c]	CCCCTGAACTGAAACCACTG
**Transcript.version (NCBI mRNA-RefSequence)**	**Convergent Primers**	**Primer sequences (5'… 3')**
NM_022073.4	linEGLN3-F[a]	CTGTCTGGTACTTTGATGCTGAA
NM_022073.4	linEGLN3-R[a]	TCAGTGAGGGCAGATTCAGTT
NM_014899.4	linRHOBTB3-F[a]	CCACCTCAACTTGAACAACCA
NM_014899.4	linRHOBTB3-R[a]	GGCAGCAGAACAGCAAGTTA
NM_001044723.2	linCSNK1G3-F[b]	TGAGAGGCAGTCTTCCTTGG
NM_001044723.2	linCSNK1G3-R[b]	ACATAACGAAGATATGTTGCCATT
NM_021130.5	PPIA-F+R mix	QuantiTect Primer Assay (QT00052311), Qiagen
NM_003194.5	TBP-F[d]	TTCGGAGAGTTCTGGGATTGTA
NM_003194.5	TBP-R[d]	TGGACTGTTCTTCACTCTTGGC
NM_000688.6	ALAS1-F[e]	GAAATGAATGCCGTGAGGAA
NM_000688.6	ALAS1-R[e]	CCTCCATCGGTTTTCACACT
NM_000194.3	HPRT1-F[e]	TGATAGATCCATTCCTATGACTGTAGA
NM_000194.3	HPRT1-R[e]	AAGACATTCTTTCCAGTTAAAGTTGAG

[a] According to Franz et al. [27].
[b] According to Chen et. [30].
[c] According to Memczak et al. [33].
[d] According to Jung et al. [28].
[e] According to Ohl et al. [29] with changed HPRT1-R primer sequence at the 3'- and 5'-ends.

Protocols for all LightCycler runs in SYBR Green and Probe assay format

qPCR reaction mix for all SYBR Green assays

Volume (µL)	Reagent/Sample	Components
5	Maxima SYBR Green qPCR Master Mix (2X)	Maxima Hot Start Taq DNA Polymerase, dNTPs (also dUTP) and SYBR Green I in an optimized PCR buffer
2	Primer Mix[a]	Forward and reverse primer mix, final concentration each 0.250 µM
1	cDNA[b]	Un- or prediluted
2	Water, nuclease free	
**Total volume 10 µL**		

[a] For PPIA qPCR, F+R primer mix included in QuantiTect Primer assay (Qiagen) was used; all other qPCR primers were synthesized by TIB Molbiol.
[b] Undiluted cDNA input for qPCR of circCSNK1G3 and linCSNK1G3 and 1:10 prediluted cDNA input for qPCR of circ- and linEGLN3, circ- and linRHOBTB3, PPIA and TBP.

LightCycler 480 SYBR Green assay run templates

**Setup**		
Block type	Reaction volume (µL)	
96	10	
Detection format	Excitation filter	Emission filter
SYBR Green	483 nm	533 nm
**Programs**		
Program names	Cycles	Analysis mode
Pre-incubation	1	None
Amplification	45	Quantification
Melting curve	1	Melting curve
Cooling	1	None

The setup and programs are equal for all assays in SYBR Green detection format.

LightCycler 480 SYBR Green assay run template for circEGLN3

**Temperature targets**					
	Target (°C)	Acquisition mode	Hold time (s)	Ramp rate (°C/s)	Acquisitions (per °C)
Pre-incubation	95	None	600	4.4	-
Amplification	95	None	15	4.4	-
	60	None	30	2.2	-
	79	Single	2	4.4	-
Melting curve	95	None	5	4.4	-
	65	None	60	2.2	-
	95	Continuous	-	0.11	5
Cooling	40	None	30	1.5	-

LightCycler 480 SYBR Green assay run template for linEGLN3, linCSNK1G3, lin- and circRHOBTB3 qPCR

**Temperature targets**					
	Target (°C)	Acquisition mode	Hold time (s)	Ramp rate (°C/s)	Acquisitions (per °C)
Pre-incubation	95	None	600	4.4	-
Amplification	95	None	15	4.4	-
	60	None	15	2,2	-
	70	Single	15	4.4	-
Melting curve	95	None	5	4.4	-
	65	None	60	2.2	-
	95	Continuous	-	0.11	5
Cooling	40	None	30	1.5	-

LightCycler 480 SYBR Green assay run template for circCSNK1G3

Temperature targets					
	Target (°C)	Acquisition mode	Hold time (s)	Ramp rate (°C/s)	Acquisitions (per °C)
Pre-incubation	95	None	600	4.4	-
Amplification	95	None	10	4.4	-
	60	None	30	2.2	-
	72	Single	2	4.4	-
Melting curve	95	None	5	4.4	-
	65	None	60	2.2	-
	95	Continuous	-	0.11	5
Cooling	40	None	30	1.5	-

LightCycler 480 SYBR Green assay run template for reference genes PPIA and TBP

Temperature targets					
	Target (°C)	Acquisition mode	Hold time (s)	Ramp rate (°C/s)	Acquisitions (per °C)
Pre-incubation	95	None	900	4.4	-
Amplification	95	None	15	4.4	-
	58	None	20	1.0	-
	72	None	20	4.4	
	79	Single	2	4.4	-
Melting curve	92	None	5	4.4	-
	65	None	60	2.2	-
	95	Continuous	-	0.11	5
Cooling	40	None	30	1.5	-

qPCR reaction mix for Probe assays for the reference genes ALAS1 and HPRT1

Volume (µL)	Reagent/Sample	Components
5	2x LightCycler 480 Probes Master	Mix containing FastStart Taq DNA Polymerase, reaction buffer, dNTP mix (with dUTP instead of dTTP), and 6.4 mM $MgCl_2$
1	Primer Mix	Forward and reverse primer mix, final concentration each 0.250 µM
1	Probe	Roche UPL Probe #40 for ALAS1 or #22 for HPRT1, final concentration 0.2 µM
1	cDNA	Undiluted
2	Water, nuclease free	
**Total volume 10 µL**		

LightCycler 480 Probe assay run template for the reference genes ALAS1 and HPRT1

Setup					
Block type			Reaction volume (µL)		
96			10		
Detection format		Excitation filter		Emission filter	
Mono Color Hydrolysis Probe		483 nm		533 nm	
**Programs**					
Program names		Cycles		Analysis mode	
Pre-incubation		1		None	
Amplification		45		Quantification	
Cooling		1		None	
**Temperature targets**					
	Target (°C)	Acquisition mode	Hold time (s)	Ramp rate (°C/s)	Acquisitions (per °C)
Pre-incubation	95	None	600	4.4	-
Amplification	95	None	10	4.4	-
	60	None	30	2.2	-
	72	Single	1	4.4	-
Cooling	40	None	30	1.5	-

3.2.2. Quantification of miRNAs

TaqMan MiRNA Assays (Applied Biosystems) were used for the detection of mature miRNAs let-7a-5p, miR-17-5p, and miR-210-3p (Supplemental Table 9). Technical details corresponded with analysis parameters given in our previous reports [34-37].

Supplemental Table S9. TaqMan MicroRNA Assays (Applied Biosystems; Assay name, Assay ID) for the measurement of mature miRNAs characterized by the miRBase accession number, the miRBase IDs, and the sequences in reference to the miRBase 22 version.

Assay name	Assay ID	miRBase accession no.	miRBase ID	Sequence
hsa-let-7a	000377	MIMAT0000062	hsa-let-7a-5p	UGAGGUAGUAGGUUGUAUAGUU
hsa-miR-17	002308	MIMAT0000070	hsa-miR-17-5p	CAAAGUGCUUACAGUGCAGGUAG
hsa-miR-210	000512	MIMAT0000267	hsa-miR-210-3p	CUGUGCGUGUGACAGCGGCUGA

The qPCR reaction mixture of 10 µL contained: 1 µL miRNA-specific cDNA, 5 µL TaqMan 2x Universal PCR Master Mix No AmpErase UNG, 0.5 µL gene-specific TaqMan MicroRNA real-time PCR-Assay solution (20x), and 3.5 µL nuclease-free water. Following cycling conditions were set: initial activation of Taq polymerase at 95 °C for 10 min, amplification steps: denaturation at 95 °C, 15 s, annealing/elongation at 60 °C for 1 min with fluorescence acquisition, and final cooling step at 40 °C for 1 min. All non-template controls were negative.

3.3. Performance data of the assays

Analytical specificity and characteristics of circEGLN3, circRHOBTB3, their linear counterparts, the reference genes, and the three miRNAs were already reported in our previous publication of circRNAs in ccRCC (Sanger sequencing, melting-point analysis, PCR-product specificity) or in other preceding publications [27-29, 35]. For the PCa-specific circRNA circCSNK1G3, analytical specificity data are given in the article by Chen et al. [30]. The analytical specificity of circCSNK1G3 was confirmed by the usual tests including Sanger sequencing. In Figure S2, its melting-point analysis and PCR-product specificity is shown together with those of circEGLN3 and circRHOBTB3. In Figure S3, the decreased cDNA synthesis of the circRNAs in the clinical samples with oligo(dT) primers is shown in comparison to the cDNA synthesis with random hexamer primers, indicating that the circRNAs have no poly(A) tail. For all assays used in this study, analytical PCR characteristics are presented in Table S10 and data of repeatability and reproducibility in Table S11.

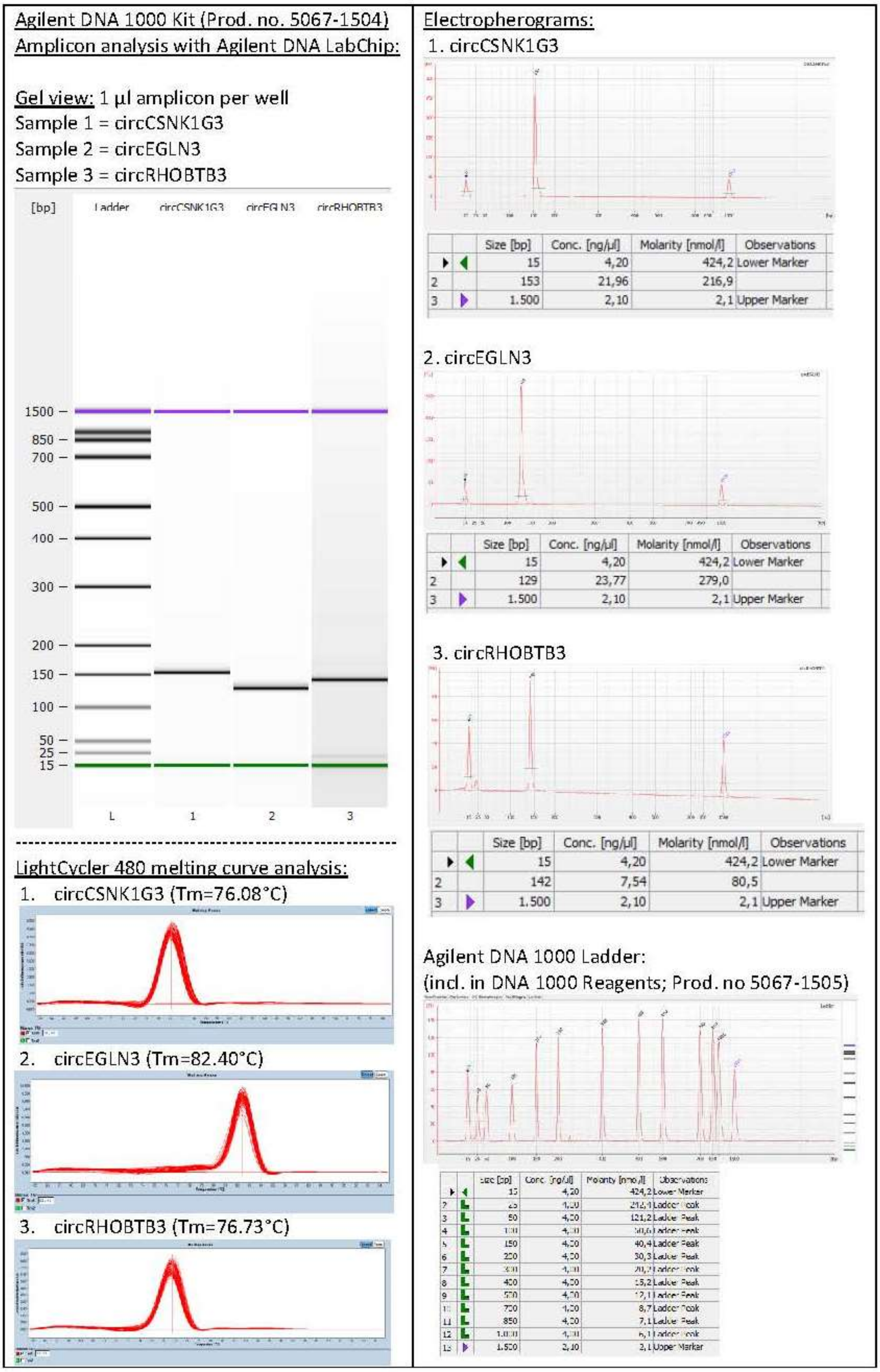

Supplemental Figure S2. Amplicon analyses of circCSNK1G3, circEGLN3, and circRHOBTB3 with Agilent Bioanalyzer 2100 (gel view and electropherogram) and with Roche LightCycler 480 Instrument (melting curve analysis). For qPCR of circCSNK1G3, published primers from Chen et al. [30] were used.

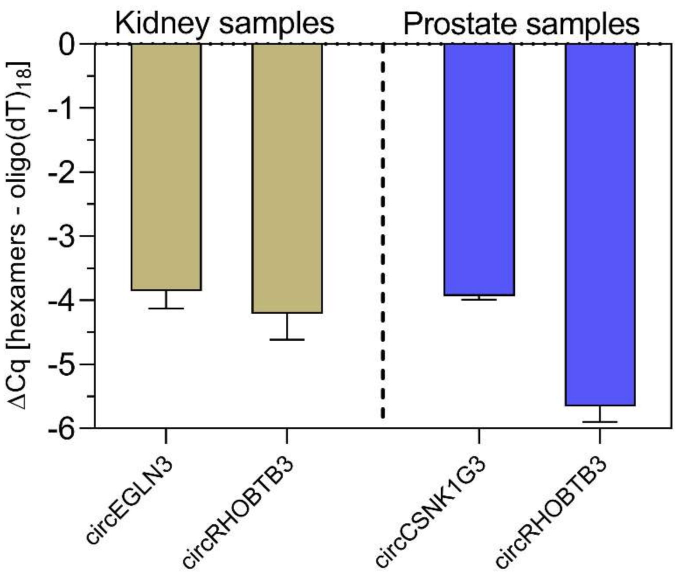

Supplemental Figure S3. Random vs. oligo(dT) primers for cDNA synthesis of circEGLN3, circCSNK1G3, and circRHOBTB3. The results of qPCR measurements showed that the relative expression was markedly decreased in all circRNAs (at least n = 3 of tissue pools) when using oligo(dT) primers in comparison to random hexamer primers, indicating that the circRNAs lack a poly(A) tail.

Supplemental Table S10. Characteristics of the qPCR standard curves.
Standard curves were generated either from diluted cDNAs or from diluted amplicons. Cq values were calculated by the LightCycler480 Software Version 1.5.1.62 using the "second derivative maximum" method. The efficiency, the slope, intercept, and error of the standard curve as well as the so-called dynamic range resulted from LightCycler480 software.

Gene	PCR efficiency[a]	Slope	y-Intercept	Error[b]	Dynamic range[c]	Cq range of samples[d]
circEGLN3	1.975	-3.385	19,75	0.0174	19.73-35.24	23.12 – 36.51 2 samples with Cq >35
circRHOBTB3[e]	2.190	-2.938	20.75	0.0466	20.96 - 32,76	23.05 - 31.13
circCSNK1G3	1.867	-3.689	21.39	0.0258	20.61 - 32.64	19.73 - 25.57 1 sample with Cq <20,61
linEGLN3[e]	1.922	-3.523	14.39	0.0112	14.40 - 34.90	17.82-31.88
linRHOBTB3	1.929	-3.504	13,50	0.0134	13.48-32.27	19.60 - 29.26
linCSNK1G3	1.958	-3.426	20.57	0.0204	20.68-34.02	20.30 – 24.55
ALAS1	1.920	-3.529	18.89	0.0118	18.89-34.18	21.08 - 25.50
HPRT1	1.863	-3.702	8.881	0.0100	9.01-31.34	23.82 - 28.42
PPIA	1.928	-3.509	16.13	0.0472	16.08-33.45	17.48 - 22.18
TBP[e]	1.840	-3.777	22.51	0.000786	22.20 - 32.20	22.02 - 28.40
let-7a-5p	1.864	-3.698	22.74	0.00602	21.60 - 33.76	21.53 - 26.19
miR-17-5p	1.871	-3.676	12.35	0.00698	12.29 - 34.96	22.81 - 25.87
miR-210-3p	1.968	-3.400	22.97	0.0647	21.92 - 38.50	24.27 - 24.78

[a] PCR efficiency is calculated by the LightCycler480 software using the equation: Efficiency=$10^{-1/slope}$.
[b] The error value is the mean squared error of the single data points fit to the regression line according to the LightCycler 480 operator's manual.
[c] Dynamic range represents the range of Cq values between the highest and the lowest Cq value of the generated standard curve.
[d] Cq range of the measured samples represents the lowest and highest Cq value measured in all samples of the degradation experiments and in the 118 clinical samples.
[e] The qPCR standard curves of circRHOBTB3, linEGLN3, and TBP were the same as previously reported [27].

Supplemental Table S11. Repeatability and reproducibility of RT-qPCR measurements.

RNA variables	Repeatability[a]			Reproducibility[b]		
	n	Cq values Mean (%RSD)	Relative quantities Mean (%RSD)	n	Cq values Mean ± SD (%RSD)	Relative quantities Mean ± SD (%RSD)
circEGLN3	20	26.80 (0.44)	1.879 (8.83)	6	30.56 ± 0.21 (0.68)	0.957 ± 0.092 (9.61)
circRHOBTB3	20	24.90 (0.37)	1.420 (6.35)	4	24.73 ± 0.14 (0.56)	1.018 ± 0.098 (9.63)
circCSNK1G3	20	22.12 (0.41)	1.321 (6.56)	5	21.53 ± 0.17 (0.89)	1.291 ± 0.160 (12.4)
linEGLN3	20	22.83 (0.28)	2.995 (6.16)	6	23.83 ± 0.17 (0.71)	1.006 ± 0.123 (12.2)
linRHOBTB3	20	21.08 (0.38)	1.187 (5.61)	5	20.51 ± 0.20 (0.97)	1.438 ± 0.198 (13.8)
linCSNK1G3	20	22.17 (0.36)	1.266 (5.31)	5	21.04 ± 0.17 (0.82)	1.979 ± 0.241 (12.2)
ALAS1	20	23.66 (0.26)	1.154 (4.19)	4	22.74 ± 0.11 (0.50)	1.675 ± 0.147 (8.78)
HPRT1	20	25.88 (0.41)	1.441 (7.30)	4	23.86 ± 0.12 (0.50)	3.404 ± 0.284 (8.34)
PPIA	20	19.36 (0.34)	1.062 (4.78)	7	19.16 ± 0.11 (0.57)	1.003 ± 0.113 (7.88)
TBP	20	27.27 (0.39)	1.360 (7.53)	7	24.92 ± 0.16 (0.64)	1.005 ± 0.113 (11.2)
let-7a-5p	20	23.22 (0.49)	1.177 (7.92)			
miR-17-5p	15	24.31 (0.26)	1.384 (4.54)			
miR-210-3p	20	23.87 (0.27)	1.361 (4.56)			

[a] Using the root mean square method, %RSD values were calculated from duplicate measurements of the Cq values and relative quantities, respectively. Relative quantities were calculated using the $2^{-\Delta\Delta Cq}$ approach with qbase^{+} software.

[b] Interassay controls; %RSD of Cq values corresponds to the percent relative standard deviation of the interassay controls. %RSD of relative quantities corresponds to the percent relative standard deviation calculated on basis of relative quantities using the $2^{-\Delta\Delta Cq}$ approach.

Cq: quantitation cycle; %RSD: percent relative standard deviation; SD: standard deviation.

4. Statistical data regarding the relationship of expression results to RIN values

Table S12. *P*-values of the expression data calculated as relative quantities and normalized quantities in cancer samples classified by the RIN limits 7 or 6. Values for the groups of RIN 6 are indicated in Figure 4.

	**Renal cell carcinoma**			
**RNA**	**Samples with RIN ≤7 and >7 (n=39/n=22)**		**Samples with RIN ≤6> and >6 (n=28/n=33)**	
	P-values between the two RIN groups within the quantifications		*P*-values between the two RIN groups within the quantifications	
	Relative quantities	Normalized quantities	Relative quantities	Normalized quantities
circEGLNN3	<0.0001	0.006	<0.0001	<0.0001
linEGLN3	<0.0001	0.0272	<0.0001	<0.0004
circRHOBTB3	0.0003	0.3595	<0.0001	0.2122
linRHOBTB3	0.0252	0.5434	0.0052	0.7473
	**Prostate cancer**			
**RNA**	**Samples with RIN ≤7 and >7 (n=38/n=19)**		**Samples with RIN ≤6> and >6 RIN limit ≤6> (n=26/n=31)**	
	P-values between the RIN groups within the quantifications		*P*-values between the RIN groups within the quantifications	
	Relative quantities	Normalized quantities	Relative quantities	Normalized quantities
circCSNK1G3	0.0104	0.8208	0.0021	0.6390
linCSNK1G3	<0.0001	0.1384	<0.0001	0.3411
circRHOBTB3	<0.0001	0.2611	<0.0001	0.0976
linRHOBTB3	<0.0001	0.1274	<0.0001	0.1173

Table S13. Regression line analysis of circRNAs and their linear counterparts calculated as relative quantities (RQ) and normalized relative quantities (NRQs) in relation to the RIN values of total RNA samples from kidney cancer in Figure 5A.

Quantification of RNA variable (RQ, NRQ)	Regression line equation	95% CI of slopes	Is slope significantly non-zero? (*P*-value)	Are the slopes equal? (*P*-value)
**circEGLN3**				
RQ (2.3-9.4)	Y = 0.9455*X - 2.188	0.5431 to 1.348	<0.0001	<0.0001
NRQ (2.3-9.4)	Y = 0.4433*X + 0.1145	0.1120 to 0.7746	0.0096	
RQ (6.1-9.4)	Y = -0.2766*X + 7.750	-1.564 to 1.011	0.6644	0.7462
NRQ (6.1-9.4)	Y = -0.5372*X + 8.053	-1.545 to 0.4703	0.2852	
**linEGLN3**				
RQ (2.3-9.4)	Y = 0.6381*X - 1.606	0.4427 to 0.8335	<0.0001	0.0041
NRQ (2.3-9.4)	Y = 0.2615*X + 0.2944	0.09397 to 0.4291	0.0028	
RQ (6.1-9.4)	Y = 0.2046*X + 1.969	-0.4299 to 0.8391	0.5156	0.4644
NRQ (6.1-9.4)	Y = -0.08939*X + 3.176	-0.6001 to 0.4214	0.7236	
**circRHOBT3**				
RQ (2.3-9.4)	Y = 0.3908*X - 0.7319	0.1925 to 0.5891	0.0002	0.0093
NRQ (2.3-9.4)	Y = 0.07253*X + 0.9296	-0.06392 to 0.2090	0.2919	
RQ (6.1-9.4)	Y = 0.04727*X + 2.055	-0.6825 to 0.7770	0.8958	0.7929
NRQ (6.1-9.4)	Y = -0.06250*X + 2.025	-0.4961 to 0.3711	0.7708	
**linRHOBTB3**				
RQ (2.3-9.4)	Y = 0.3033*X - 0.2363	0.1124 to 0.4941	0.0023	0.0159
NRQ (2.3-9.4)	Y = 0.000459*X + 1.426	-0.158 to 0.159	0.9954	
RQ (6.1-9.4)	Y = 0.02537*X + 1.964	-0.6619 to 0.7126	0.9405	0.7489
NRQ (6.1-9.4)	Y = -0.08375*X + 2.012	-0.5157 to 0.3482	0.6952	

RQ: relative quantification; NRQ: normalized relative quantification using the reference genes PPIA and TBP. The numbers in brackets (2.3-9.4) and (6.1-9.4) refer to the total RNA samples with the ranges of RIN values used for relative or normalized relative quantification.

Supplemental Table S14. Regression line analysis of circRNAs and their linear counterparts calculated as relative and normalized quantities in relation to the RIN values of total RNA samples from prostate cancer in Figure 5B.

RNA variables	Regression line equation	95% CI of slopes	Is slope significantly non-zero? (*P*-value)	Are the slopes equal? (*P*-value)
**circCSNK1G3**				
RQ (2.2-8.2)	Y = 0.1689*X + 0.2721	0.06446 to 0.2734	0.0020	0.0491
NRQ (2.2-8.2)	Y = 0.04611*X + 0.8797	-0.03305 to 0.1253	0.2481	
RQ (6.3-8.2)	Y = 0.1673*X + 0.2970	-0.4058 to 0.7404	0.5551	0.4206
NRQ (6.3-8.2)	Y = -0.09948*X + 1.871	-0.4519 to 0.2529	0.5682	
**linCSNK1G3**				
RQ (2.2-8.2)	Y = 0.1583*X + 0.2697	0.08914 to 0.2274	<0.0001	0.0015
NRQ (2.2-8.2)	Y = 0.03401*X + 0.8372	0.000987 to 0.06704	0.0438	
RQ (6.3-8.2)	Y = 0.4374*X - 1.712	0.1384 to 0.7364	0.0056	0.0702
NRQ (6.3-8.2)	Y = 0.1067*X + 0.3027	-0.05119 to 0.2645	0.1775	
**circRHOBT3**				
RQ (2.2-8.2)	Y = 0.2821*X - 0.2899	0.2084 to 0.3557	<0.0001	0.0160
NRQ (2.2-8.2)	Y = 0.1423*X + 0.4346	0.05470 to 0.2300	0.0019	
RQ (6.3-8.2)	Y = 0.5899*X - 2.537	0.2227 to 0.9571	0.0027	0.0808
NRQ (6.3-8.2)	Y = 0.1244*X + 0.4702	-0.2659 to 0.5146	0.5196	
**linRHOBTB3**				
RQ (2.2-8.2)	Y = 0.2200*X + 0.01879	0.1336 to 0.3063	<0.0001	0.0089
NRQ (2.2-8.2)	Y = 0.08193*X + 0.6510	0.02423 to 0.1396	0.0062	
RQ (6.3-8.2)	Y = 0.5374*X - 2.267	0.1721 to 0.9027	0.0054	0.0691
NRQ (6.3-8.2)	Y = 0.1514*X + 0.1060	-0.06801 to 0.3709	0.1688	

RQ: relative quantification; NRQ: normalized relative quantification using the reference genes ALAS1 and HPRT1. The numbers in brackets (2.2-8.2) and (6.3-8.2) after RQ and NRQ refer to the total RNA samples with the ranges of RIN values used for relative or normalized relative quantification.

5. References

1. Li P, Chen S, Chen H, Mo X, Li T, Shao Y, et al. Using circular RNA as a novel type of biomarker in the screening of gastric cancer. Clin Chim Acta. 2015; 444: 132-6.
2. Ahmed I, Karedath T, Andrews SS, Al-Azwani IK, Mohamoud YA, Querleu D, et al. Altered expression pattern of circular RNAs in primary and metastatic sites of epithelial ovarian carcinoma. Oncotarget. 2016; 7: 36366-81.
3. Lu L, Sun J, Shi P, Kong W, Xu K, He B, et al. Identification of circular RNAs as a promising new class of diagnostic biomarkers for human breast cancer. Oncotarget. 2017; 8: 44096-107.
4. Zhu X, Wang X, Wei S, Chen Y, Chen Y, Fan X, et al. hsa_circ_0013958: a circular RNA and potential novel biomarker for lung adenocarcinoma. FEBS J. 2017; 284: 2170-82.
5. Dang Y, Ouyang X, Zhang F, Wang K, Lin Y, Sun B, et al. Circular RNAs expression profiles in human gastric cancer. Sci Rep. 2017; 7: 9060.
6. Weng W, Wei Q, Toden S, Yoshida K, Nagasaka T, Fujiwara T, et al. Circular RNA ciRS-7-A promising prognostic biomarker and a potential therapeutic target in colorectal cancer. Clin Cancer Res. 2017; 23: 3918-28.
7. Cao S, Wei D, Li X, Zhou J, Li W, Qian Y, et al. Novel circular RNA expression profiles reflect progression of patients with hypopharyngeal squamous cell carcinoma. Oncotarget. 2017; 8: 45367-79.
8. Chen S, Li T, Zhao Q, Xiao B, Guo J. Using circular RNA hsa_circ_0000190 as a new biomarker in the diagnosis of gastric cancer. Clin Chim Acta. 2017; 466: 167-71.
9. Huang XY, Huang ZL, Xu YH, Zheng Q, Chen Z, Song W, et al. Comprehensive circular RNA profiling reveals the regulatory role of the circRNA-100338/miR-141-3p pathway in hepatitis B-related hepatocellular carcinoma. Sci Rep. 2017; 7: 5428.

10. Zhang F, Zhao X, Dong H, Xu J. circRNA expression analysis in lung adenocarcinoma: comparison of paired fresh frozen and formalin-fixed paraffin-embedded specimens. Biochem Biophys Res Commun. 2018; 500: 738-43.
11. Sun S, Li B, Wang Y, Li X, Wang P, Wang F, et al. Clinical significance of the decreased expression of hsa_circ_001242 in oral squamous cell carcinoma. Dis Markers. 2018; 2018: 6514795.
12. Huang Y, Zhang Y, Jia L, Liu C, Xu F. Circular RNA ABCB10 promotes tumor progression and correlates with pejorative prognosis in clear cell renal cell carcinoma. Int J Biol Markers. 2019; 34: 176-83.
13. Chen J, Chen T, Zhu Y, Li Y, Zhang Y, Wang Y, et al. circPTN sponges miR-145-5p/miR-330-5p to promote proliferation and stemness in glioma. J Exp Clin Cancer Res. 2019; 38: 398.
14. Lu C, Jiang W, Hui B, Rong D, Fu K, Dong C, et al. The circ_0021977/miR-10b-5p/P21 and P53 regulatory axis suppresses proliferation, migration, and invasion in colorectal cancer. J Cell Physiol. 2020; 235: 2273-85.
15. Jin C, Dong D, Yang Z, Xia R, Tao S, Piao M. CircMYC regulates glycolysis and cell proliferation in melanoma. Cell Biochem Biophys. 2020; 78: 77-88.
16. Xing Y, Zha WJ, Li XM, Li H, Gao F, Ye T, et al. Circular RNA circ-Foxo3 inhibits esophageal squamous cell cancer progression via the miR-23a/PTEN axis. J Cell Biochem. 2020; 121: 2595-605.
17. Sun D, Chen L, Lv H, Gao Y, Liu X, Zhang X. Circ_0058124 upregulates MAPK1 expression to promote proliferation, metastasis and metabolic abilities in thyroid cancer through sponging miR-940a. OncoTargets Ther. 2020; 13: 1569-81.
18. Liu Y, Xia L, Dong L, Wang J, Xiao Q, Yu X, et al. CircHIPK3 promotes gemcitabine (GEM) resistance in pancreatic cancer cells by sponging miR-330-5p and targets RASSF1. Cancer Manag Res. 2020; 12: 921-9.
19. Zang Y, Li J, Wan B, Tai Y. CircRNA circ-CCND1 promotes the proliferation of laryngeal squamous cell carcinoma through elevating CCND1 expression via interacting with HuR and miR-646. J Cell Mol Med. 2020; 24: 2423-33.
20. Li S, Pei Y, Wang W, Liu F, Zheng K, Zhang X. Extracellular nanovesicles-transmitted circular RNA has_circ_0000190 suppresses osteosarcoma progression. J Cell Mol Med. 2020; 24: 2202-14.
21. Zhou F, Wang D, Wei W, Chen H, Shi H, Zhou N, et al. Comprehensive profiling of circular RNA expressions reveals potential diagnostic and prognostic biomarkers in multiple myeloma. BMC Cancer. 2020; 20: 40.
22. Kong Z, Wan X, Lu Y, Zhang Y, Huang Y, Xu Y, et al. Circular RNA circFOXO3 promotes prostate cancer progression through sponging miR-29a-3p. J Cell Mol Med. 2020; 24: 799-813.
23. Meng QH, Li Y, Kong C, Gao XM, Jiang XJ. Circ_0000388 exerts oncogenic function in cervical cancer cells by regulating miR-337-3p/TCF12 axis. Cancer Biother Radiopharm. 2020; Epub ahead of print, March 2, 2020; doi: 10.1089/cbr.2019.3159.
24. Wei X, Zheng W, Tian P, He Y, Liu H, Peng M, et al. Oncogenic hsa_circ_0091581 promotes the malignancy of HCC cell through blocking miR-526b from degrading c-MYC mRNA. Cell Cycle. 2020; 19: 817-24.
25. Yu Q, Liu P, Han G, Xue X, Ma D. CircRNA circPDSS1 promotes bladder cancer by down-regulating miR-16. Biosci Rep. 2020; Epub ahead of print, January 31, 2020; doi: 10.1042/BSR20191961.
26. Bustin SA, Benes V, Garson JA, Hellemans J, Huggett J, Kubista M, et al. The MIQE guidelines: minimum information for publication of quantitative real-time PCR experiments. Clin Chem. 2009; 55: 611-22.
27. Franz A, Ralla B, Weickmann S, Jung M, Rochow H, Stephan C, et al. Circular RNAs in clear cell renal cell carcinoma: their microarray-based identification, analytical validation, and potential use in a clinico-genomic model to improve prognostic accuracy. Cancers (Basel). 2019; 11: E1473.
28. Jung M, Ramankulov A, Roigas J, Johannsen M, Ringsdorf M, Kristiansen G, et al. In search of suitable reference genes for gene expression studies of human renal cell carcinoma by real-time PCR. BMC Mol Biol. 2007; 8: 47.
29. Ohl F, Jung M, Xu C, Stephan C, Rabien A, Burkhardt M, et al. Gene expression studies in prostate cancer tissue: which reference gene should be selected for normalization? J Mol Med. 2005; 83: 1014-24.
30. Chen S, Huang V, Xu X, Livingstone J, Soares F, Jeon J, et al. Widespread and functional RNA circularization in localized prostate cancer. Cell. 2019; 176: 831-43.
31. Jeck WR, Sharpless NE. Detecting and characterizing circular RNAs. Nat Biotechnol. 2014; 32: 453-61.
32. Untergasser A, Cutcutache I, Koressaar T, Ye J, Faircloth BC, Remm M, et al. Primer3 - new capabilities and interfaces. Nucleic Acids Res. 2012; 40: e115.
33. Memczak S, Jens M, Elefsinioti A, Torti F, Krueger J, Rybak A, et al. Circular RNAs are a large class of animal RNAs with regulatory potency. Nature. 2013; 495: 333-8.

34. Jung M, Mollenkopf H-J, Grimm C, Wagner I, Albrecht M, Waller T, et al. MicroRNA profiling of clear cell renal cell cancer identifies a robust signature to define renal malignancy. J Cell Mol Med. 2009; 13: 3918-28.
35. Jung M, Schaefer A, Steiner I, Kempkensteffen C, Stephan C, Erbersdobler A, et al. Robust microRNA stability in degraded RNA preparations from human tissue and cell samples. Clin Chem. 2010; 56: 998-1006.
36. Schaefer A, Jung M, Mollenkopf HJ, Wagner I, Stephan C, Jentzmik F, et al. Diagnostic and prognostic implications of microRNA profiling in prostate carcinoma. Int J Cancer. 2010; 126: 1166-76.
37. Wotschofsky Z, Meyer H-A, Jung M, Fendler A, Wagner I, Stephan C, et al. Reference genes for the relative quantification of microRNAs in renal cell carcinomas and their metastases. Analyt Biochem. 2011; 417: 233-41.
